# Impact of an intervention program on drug adherence in patients with ulcerative colitis: Randomized clinical trial

**DOI:** 10.1371/journal.pone.0295832

**Published:** 2023-12-27

**Authors:** Mila Pacheco, Pedro Sá, Gláucia Santos, Ney Boa-Sorte, Kilma Domingues, Larissa Assis, Marina Silva, Ana Oliveira, Daniel Santos, Jamile Ferreira, Rosemeire Fernandes, Flora Fortes, Raquel Rocha, Genoile Santana

**Affiliations:** 1 Departamento de Ciências da Vida, Universidade do Estado da Bahia (UNEB), Salvador, Bahia, Brazil; 2 Centro de Infusões e Medicamentos Especializados da Bahia (CIMEB), Salvador, Bahia, Brazil; 3 Universidade Federal da Bahia (UFBA), Canela, Salvador, BA–Brazil; Kaohsiung Medical University, TAIWAN

## Abstract

**Aims:**

Evaluate the impact of an intervention program in non-adherent patients with ulcerative colitis.

**Methods:**

Parallel controlled randomized clinical trial (1:1), approved by the ethics committee (No. 3.068.511/2018) and registered at The Brazilian Clinical Trials Registry (No. RBR-79dn4k). Non-adherent ulcerative colitis patients according to the Morisky-Green-Levine-test were included. Recruitment began in August 2019 until August 2020, with 6-month follow-up. All participants received standard usual care, and additionally the intervention group received educational (video, educational leaflet, verbal guidance) and behavioral interventions (therapeutic scheme, motivational and reminder type short message services). Researchers were blinded for allocation prior to data collection at Visits 1 and 2 (0 and 6 months). Primary outcome: 180-day adherence rate, with relative risk 95%CI. Secondary outcome: 180-day quality of life according to SF-36 domains, using Student’s t test. Variables with p<0.20 were selected for regression. Analysis included data from August/2019 to May/2021.

**Results:**

Forty-six and 49 participants were allocated in control and intervention groups, respectively. Two were excluded due to intervention refusal, and 4 and 6 were lost to follow-up in control and intervention groups. There was no post-intervention adherence rate difference, even after adjustment for type of non-adherence (unintentional/both/intentional) as confounder, or if considered as adherent the intervention group participants lost in follow-up. Interventions promoted better quality of life scores even after multivariate analysis for “Pain”, when adjusted for ulcerative colitis severity, sex, and marital status (β = 18.352, p = 0.004), “Vitality”, when adjusted for ulcerative colitis severity (β = 10.568, p = 0.015) and “Emotional Aspects”, when adjusted for disease severity, income, and education (β = 24.907, p = 0.041).

**Conclusions:**

The intervention program was not able to produce a significant medication adherence rate difference between comparative groups, however, there was a significant improvement in quality of life. Study limitations may include: sample size calculated to identify differences of 30%, leading to a possible insufficient power; non blinded participants, exposing the results to the risk of performance bias; outcomes based on self-reported data.

## 1. Introduction

Low adherence to drug treatment has been identified as a serious public health problem with a magnitude ranging from 15% to 93% for patients with chronic diseases, with an estimated average of 50%, depending on the method applied, characterized as an "invisible epidemic" [1]. The lack of adherence generates direct and indirect costs, in addition to clinical, social and environmental repercussions, so it is important, therefore, not only to identify its magnitude, but also to plan and implement solutions to effectively address this issue [2, 3]. Special attention must be given to chronic diseases [2, 4, 5]. In view of this reality, global strategies have been presented as promising in the engagement against non-adherence. Numerous types of interventions have been studied, and systematic reviews have assessed these results [6–12]. However, few studies have aimed interventions to improve medication adherence specifically to ulcerative colitis (UC) patients [13].

Ulcerative colitis is a chronic inflammatory bowel disease that requires continuous drug treatment for its control, therefore, the lack of adherence constitutes a barrier to the achievement of therapeutic goals [3, 14]. The disease has a high negative impact on quality of life (QoL) and treatment is time consuming, maintained even in the remission phase, thus increasing the risk of non-adherence [15–18]. Uncontrolled UC is a socially limiting condition due to its embarrassing symptoms, causing absenteeism and frequent consultations for medical care, in addition to emotional and behavioral difficulties [19, 20].

Nieuwlaat *et al*. [13] updated a systematic review of the literature by Haynes *et al*. [21] on interventions with an impact on medication adherence for chronic diseases. Despite the large number of new publications that were included in this update (109 new randomized controlled trials), only 17 were at low risk of bias, and among these, 8 generated improvements to adherence rates. This systematic review could not combine results of included studies and perform meta-analysis, not obtaining a quantified estimates effect. The authors claim that the new studies’ results compared to the previous review, were lacking convincing evidence. It is noteworthy to say that only one trial with UC patients was selected for review, but this study was not classified as low risk bias. The authors concluded that the interventions that generated significant impact (although modest) are complex and, therefore, difficult to implement in the routine of health services. Strategies have been identified as potentially effective for improving adherence in patients with UC, including educational measures, telemedicine, behavioral changes and simplification of therapeutic schemes [22, 23].

In March 2020, the World Health Organization declared a pandemic, bringing with it several direct and indirect consequences. A systematic review investigated the impact on medication adherence with oral and biological medications for patients with Inflammatory Bowel Diseases (IBD) in this period. Major or minor impacts on adherence to treatment were identified for patients with IBD according to geographic regions. In addition, the result may have varied as a result of what each study considered as low or high adherence and the methods for measuring it. The results point to the following reasons for non-adherence: concern of patients and the health team regarding the safety of the treatment (risk of infections and adverse effects) and difficulties in accessing health services and medication due to social distancing and lockdown [24].

The aim of the present study was to evaluate the impact of an intervention program on adherence rates and QoL in patients with UC who do not adhere to drug treatment.

## 2. Materials and methods

The protocol for this trial and CONSORT checklist are available as supporting information; see S2 and S3 Files.

### 2.1. Study location

The study was carried out at a public outpatient pharmacy unit (POP) responsible for dispensing medications to patients with different health conditions, including patients with IBD. The study site was in the city of Salvador, capital of the state of Bahia, Brazil. At that time UC patients had access exclusively to oral (mesalamine, sulfasalazine, azathioprine, cyclosporine) and rectal treatment (mesalamine). In 2019, the unit had 855 patients with UC registered for care, including 590 users assisted by physicians from the public health system and 265 from the complementary health system.

### 2.2. Study design

It was a randomized, pragmatic parallel group clinical trial with two arms (1:1), in which attention was paid to preserve routine conditions of care and service.

The research protocol was registered in the Brazilian Registry of Clinical Trials with primary identifier number RBR-79dn4k.

### 2.3. Target population

The target population were patients diagnosed with UC in the state of Bahia assisted by physician from public system and pharmacist assistance from POP. Routinely, patients returned monthly to acquire their medications. However, due to the pandemic caused by the 2019 new coronavirus disease (COVID-19), there was an effort to assist them with a greater amount of medication, to reduce exposure and contribute to social distancing.

### 2.4. Inclusion criteria

The study included patients diagnosed with UC who, during the recruitment phase, irrespective of the disease activity met all the following criteria: had at least three months of active registration at POP; prescribed with at least one medication to treat UC; aged 18 or over; able to answer the research instruments; who were assisted by the IBD outpatient clinics at two reference centers; who had a cell phone; and were classified as non-adherent according to Morisky-Green-Levine Test (MGLT).

### 2.5. Exclusion criteria

Participants who were adherent according to MGLT. Lost to follow-up was defined as three unsuccessful attempts to contact the participant via phone calls in different working days occurred and/or there was refusal to be interviewed within the Visit 2 interval period.

### 2.6. Recruitment

**Fig 1:**
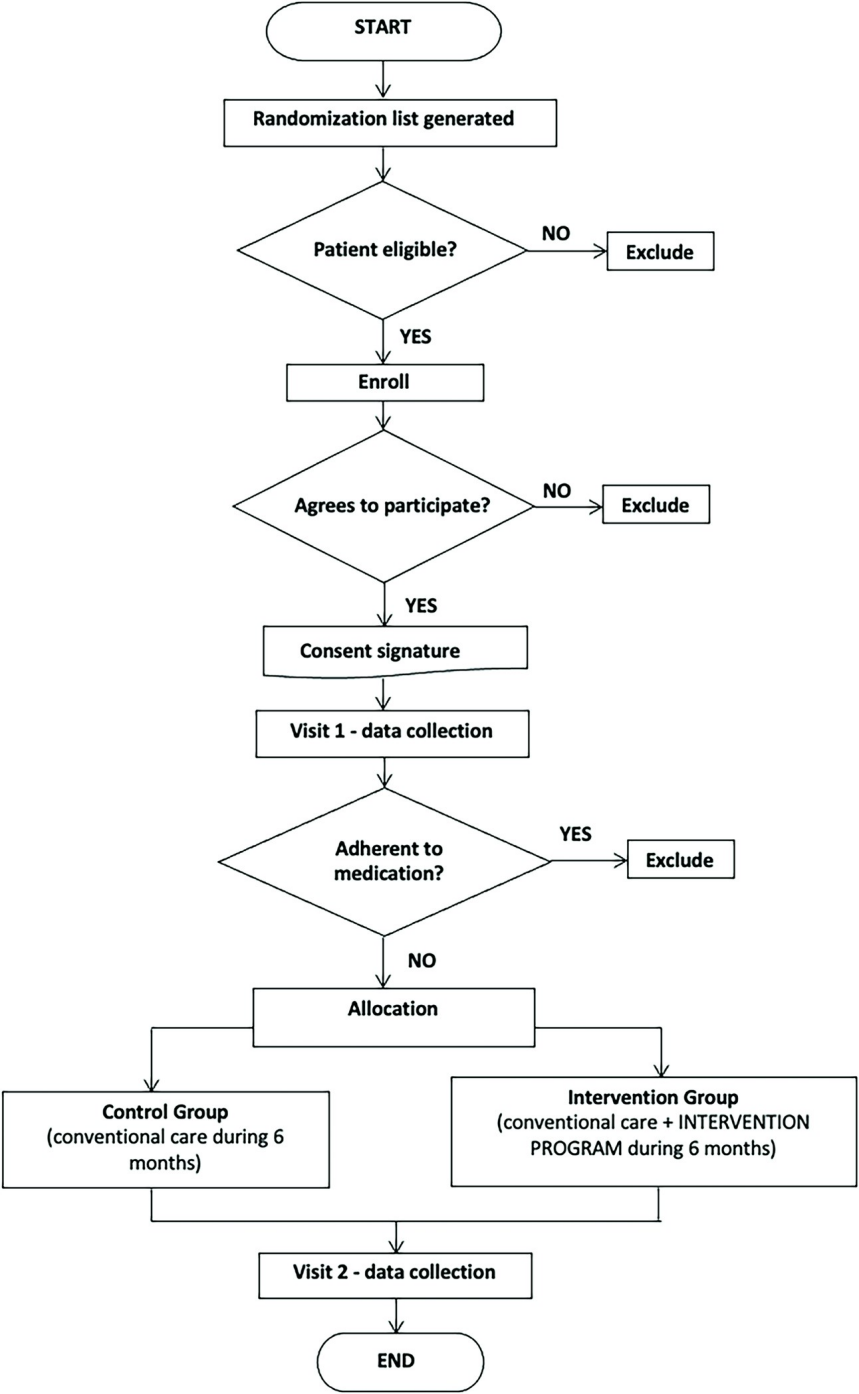
Trial flow diagram.

The study started with the recruitment stage since August 2019 until August 2020, while follow-up began at February 2020 until March 2021. The identification of patients eligible to participate in the research was carried out as they presented themselves at POP, according to information contained in their medical records. Then, the service user was approached by an investigator to identify whether it was the patient himself, as well as to present the research and confirm his eligibility. See Fig 1 for a flow diagram of the trial.

According to the sample calculation, number of interviewers and flow of patients with UC at POP, a period of recruitment of 4 months was initially estimated. However, there was an extension of this period, since there was a significant percentage of patients’ representatives. Next, COVID-19 pandemic status was declared, which also contributed to reduce the number of patients presented at the pharmacy.

### 2.7. Randomization

The simple randomization process was used, generating a list of 108 allocation positions prior to participants recruitment through an online tool (www.randomization.com). To guarantee allocation positions confidentiality, such information was kept in opaque envelopes, individually sealed, and sequentially numbered. The 1: 1 ratio between the allocation groups was used and the unit of randomization was per participant.

### 2.8. Participant’s allocation

After signing the Informed Consent Form (ICF), participants were interviewed in person and if they were considered adherent to drug treatment for UC according to MGLT, the interview was interrupted, and they were not allocated. Non-adherent respondents were fully interviewed on visit 1, and then allocated according to information contained in the numbered envelope and opened consecutively, resulting from the randomization process. This procedure aimed to reduce interviewer’s bias during data collection at visit 1. Participants were allocated to the Intervention Group (IG) or Control Group (CG) and the allocation secrecy was preserved, as the researchers who performed the interventions were different from those who measured the outcomes. Both CG and IG received the standard care offered at POP, and the IG was also exposed to the intervention program (see S1 File).

### 2.9. Data collection

Data collection was carried out by qualified interviewers that attended to the Good Clinical Practices course (https://gcp.nidatraining.org/). Structured interview with questionnaires designed specifically for this study were used to collect data on visit 1, prior to the proposed intervention program, and on visit 2, 180 days after application of the intervention program.

Instruments validated in the Brazilian reality were used, such as the MGLT [25] that measures the degree of adherence. For this research, the concept of “adherence” was considered, as:

“The extent to which a person’s behavior ‐ taking medication, following a diet and / or making lifestyle changes ‐ corresponds to the recommendations agreed with a caregiver” [2, 26].

The MGLT has four questions, and those who answered “no” to all questions were considered adherent, whereas those who answered “yes” to at least one question, were classified as non-adherent. When one or two questions were answered affirmatively, the respondent’s behavior was classified as moderately adherent. An affirmative answer to three or four questions was classified as having low adherence. When answering affirmatively to the first two questions of the MGLT, the behavior was considered “unintentional”, when answering affirmatively to the last two questions of the MGLT, the behavior was considered “intentional” [27].

For participants’ health-related QoL assessment, the Brazil Short Form Health Survey 36 (SF-36) was used [28].

The Lichtiger Index [29] was used to determine the clinical activity of UC, based on self-reported data by the participant.

### 2.10. Blinding

The participants received a unique code to identify all the instruments and forms of the research, with the omission of their names. The blinding of those involved was difficult, since the application of the intervention program automatically reveals to the participants in which group, they were allocated, making masking impossible.

The research team was divided into the following dedicated activities: a) recruitment, data collection at visit 1, allocation and execution of the intervention program for the IG (non-blind researchers); b) database feeding (non-blind researchers); c) data collection at visit 2 (blind researcher); d) analysis of the database (non-blind researchers).

When participants entered the survey and prior to the final data collection, they were asked not to disclose to other participants or the research team which group they had been allocated to.

### 2.11. Intervention program

The standard of usual care at POP has been carried out as follows: all patients who would start treatment were consulted by a pharmacist to assess the presence of contraindications, drug interactions and aspects that have relevant interference in drug treatment. Patients were instructed on how to use the medications, storage conditions and possible adverse effects. During the next attendance to POP the patient could receive medication from a technical assistant. In case of doubts, adverse events, suspension/exchange of medication, or altered exam results, these patients were referred to a new pharmaceutical consultation.

The intervention program of the present research was applied only to the participants allocated in the IG, individually in a private environment, consisting of educational interventions (video, educational leaflet, verbal guidance) and behavioral interventions (therapeutic scheme, motivational and reminder type short message services), following previously established frequencies, as explained below.

Educational Interventions:

- A video lasting about 5 minutes was played, presenting basic content about IBD and the importance of adherence to prescribed drug treatment, prepared by the research team according to previous script and text (see S1 File and Appendix 6 and 7). The video is available at the following link: link: https://youtu.be/vcvm9DXAXNg. This intervention was carried out at Visit 1;
- A printed educational leaflet (see S1 File and Appendix 8) was delivered, presenting basic contents about UC and the importance of adherence to prescribed drug treatment, prepared by the research team based on available scientific evidence, according to pre-established script (see S1 File and Appendix 9). The participants had 10 minutes for silent reading, and at the end, the team was available to answer participants’ questions. This intervention was carried out at Visit 1;
- Participants were oriented on the drugs prescribed for UC available for dispensing at POP. Verbal guidelines were made following a script (see S1 File and Appendix 11). This intervention was carried out: at Visit 1, or when medication changed during study period, or when participant had doubts about medication;

Behavioral Interventions:

- a therapeutic regimen was prepared and delivered to participant (see S1 File and Appendix 12). Participants who demonstrated difficulties in understanding the written guidelines additionally received a Pharmaceutical Guidance Table (see S1 File and Anexx A), in order to facilitate the understanding of the times and quantities of each prescribed drug. This intervention was carried out: at Visit 1; or when medication changed during study period; or when participant had doubts about the therapeutic regimen;
- Short Message Service (SMS) messages were forwarded to the cell phones registered by the participants:
- reminder messages of every service date for acquiring medication at POP). This intervention was carried out between at least 2 days before the return date and 2 hours before the scheduled time during study period (6 months);
- motivational messages were sent (see S1 File and Appendix 14). This intervention was carried out once a week during study period (6 months).

### 2.12. Data categorization

Sociodemographic and economic variables (sex, age, ethnicity/color, origin, marital status, monthly income, education) and pharmacotherapeutic variables (drugs in use for UC and comorbidities, number of dosage units in use for the treatment of UC, UC treatment schemes, medication adherence of drugs for UC, according to the MGLT [25]. The disease activity was analyzed according to LI [29], for which the score varies from 0 to 21 (higher score means worse disease activity). Quality of life was measured using the eight domains of the Brazil SF-36 questionnaire [28], for which the higher the score, the better QoL [30].

### 2.13. Sample calculation and selection

Based on the results of a study published in Brazil [31], the sample size was calculated for the primary outcome of adherence, in which a 95% bilateral significance level (1-alpha) was adopted, (1-beta) with 80% probability of detection, sample size ratio between exposed/unexposed of 1, positive unexposed percentage of 50%, positive exposed percentage of 80% and risk ratio of 1.6. The calculation was done using OpenEpi®, version 3.01, available at www.openepi.com, and resulted in a total sample size of 90 UC patients, 45 participants in each group (CG and IG). Twenty percent was added to the sample size, predicting losses to follow-up, resulting in a final intended sample size of 108.

Due to the difficulties already reported for recruitment, the research team decided to end the recruitment stage in September 2020.

#### 2.14. Endpoints

The primary endpoint was pre-specified as adherence to medication for UC according to MGLT.

The QoL scores were pre-specified as secondary outcomes according to the SF-36 Brazil instrument.

### 2.15. Statistical analysis

Data was entered into a database built with the Statistical Package for the Social Sciences®, version 21.0. The analysis plan was finalized and approved prior to the data lock for the final analysis. Quantitative variables were described as means (standard deviation), and non-normal quantitative variables were summarized by median (IQR). Qualitative variables were described with measures of absolute and percentage frequency. To compare intervention and control groups in relation to qualitative variables, Pearson’s chi-square test or Fischer’s exact test was used, when recommended. The comparison of quantitative variables between groups was performed by applying the Student’s t test for unpaired samples or ANOVA, if indicated, the non-parametric Mann-Whitney test or Kruskal Wallis test.

The occurrence of the primary study outcome (adherence) was calculated by the ratio between the total number of participants who achieved adherence to treatment and the total number of participants in the group, multiplied by 100, calculated separately for the intervention and comparative groups, in times 0 and 6 months. The calculation was carried out either considering maximum adherence (those at baseline who had low or moderate adherence and after 6 months they became adherent) or improvement in adherence (those who at baseline had low adherence and after 6 months became adherent or reached moderate adherence, as well as who in the baseline had moderate adherence and after 6 months became adherent).

For primary outcomes, the relative risk with the respective 95% CI was used as a measure of effectiveness, obtained from the ratio between the adherence of the intervention group and the adherence of the comparative group, at times 0 and 6 months after application of the intervention program. For adjustments for potential confounders, Logistic Regression was used to estimate the adjusted ORs with the respective 95% CI. Associations with p values less than 0.05 were considered significant. The analysis was made per protocol.

The analysis of QoL (secondary outcome) was performed by comparing the mean differences of the 8 domains of the SF-36 score between Visit 2 and Visit 1 with the paired t-test calculated, separately for the CG and IG. The variables that showed differences of p <0.20 were included in the construction of the regression models. For adjustments for potential confounders, Multiple Linear Regression was used to adjust the effect of the intervention by confounding variables.

### 2.16. Ethical considerations

The study was approved at December 2018 by the Research Ethics Committee of the Universidade do Estado da Bahia (No. 3.068.511/2018). The written ICF was presented and signed by all participants. All study procedures followed Brazilian ethical legislation and the trial has been sent to registry at The Brazilian Clinical Trials Registry (REBEC) since February 2019. All adjustments and information required by REBEC had no ethical or methodological implications and were made accordingly. The approval was updated online as January 2020 (No. RBR-79dn4k).

The COVID-19 pandemic was declared in March 11^th^ 2020, and considered an extenuating circumstance, since it was an unavoidable external event that would prevent trial participants from adhering to the trial protocol. At this point, 84 participants were enrolled and only 10 had completed the trial. Therefore, the following modifications were planned and reviewed to the initial protocol by the research team to mitigate possible impacts and an amendment was approved by the Research Ethics Committee:

- Expansion of the data collection window on Visit 2 to 1 month before and 1 month after the 180 days counted from the date of Visit 1;
- Optional conduction of final interview (Visit 2) via phone call, registered on a file containing telephone number, date and time of interview. This modification was important to preserve isolation during peak phases of the virus dissemination, while maintaining the study’s feasibility.

## 3. Results

**Fig 2:**
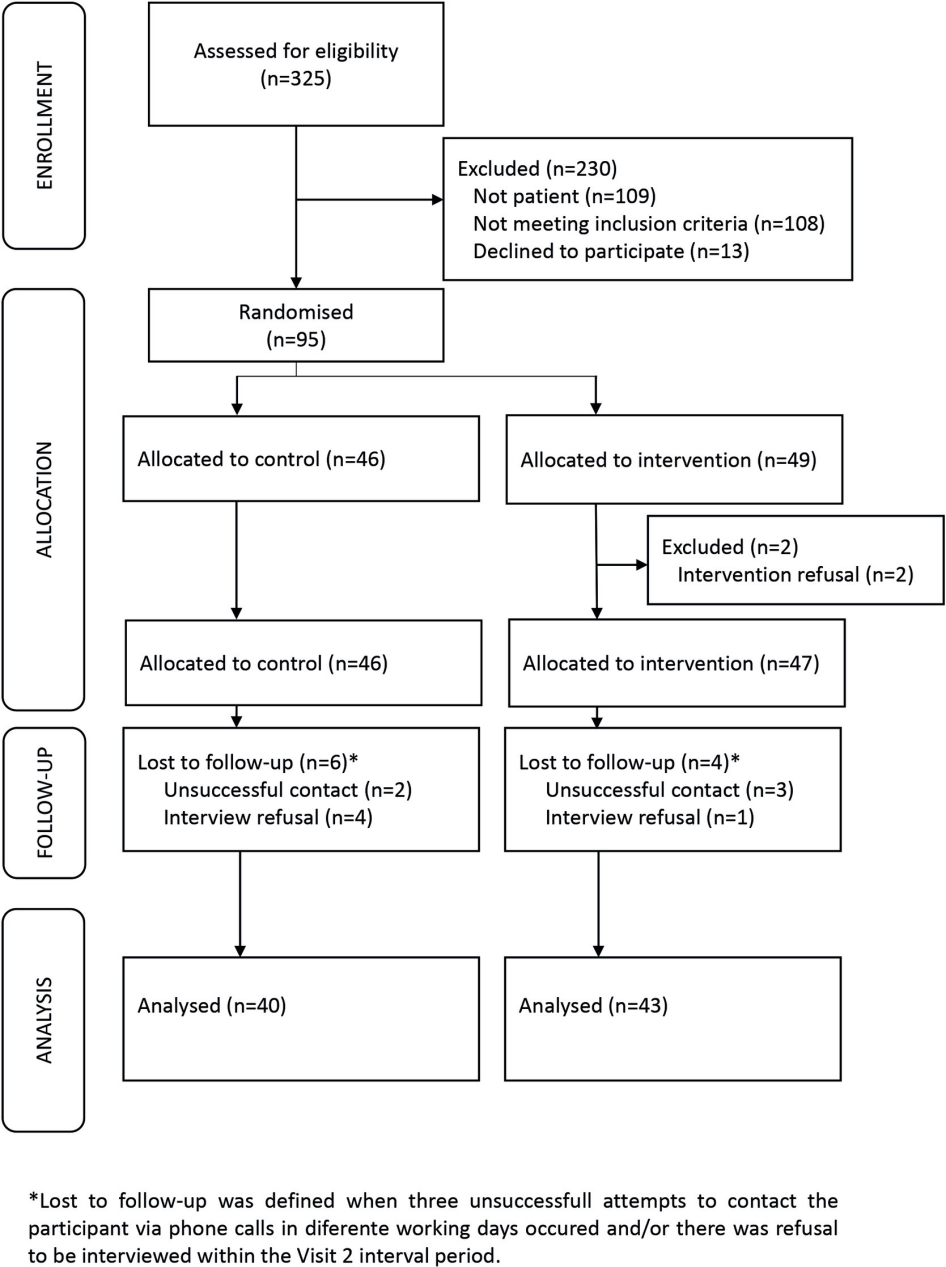
Selection, recruitment and allocation of research participants, Salvador, BA (2019–2021).

During the study period, 325 approaches were made to POP users considered for eligibility, and 95 were allocated to the research from August 2019 to October 2020, randomly assigned to groups CG and IG, 46 and 49, respectively. Two were excluded due to intervention refusal, and 4 and 6 were lost to follow-up in control and intervention groups Eighty-three participants completed the research protocol, thus, the final sample analyzed was 40 in the CG and 43 in the IG (Fig 2).

### 3.1. Participants baseline characteristics

Socio-demographic and economic baseline participants’ characteristics, distributed by allocation group are shown at Table 1.

**Table 1:** Distribution of social, demographic, and economic variables according to group allocation at baseline, Salvador, BA (2019–2021).

Variables	CG n = 46 (%)	IG n = 47 (%)	Total n = 93 (%)
**Age (yr)** ^**a**^	45.5(12.0)	48.3(12.5)	46.9(12.3)
**Children per family** ^**a**^	2.0 (1.8)	1.8 (1.5)	1.9 (1.6)
**BMI** ^**a**^	25.3 (4.9)	26.1 (5.2)	25.7 (5.0)
**Sex** Male Female	16 (34.8) 30 (65.2)	10 (21.3) 37 (78.7)	26 (28.0) 67 (72.0)
**Origin** Countryside Urban	7 (15.2) 39 (84.8)	3 (6.4) 44 (93.6)	10 (10.8) 83 (89.2)
**Skin color** Black Non-black White Mixed race Yellow Indigenous	19 (41.3) 27 (58.7) 5 (10.9) 21 (45.7) - 1 (2.2)	24 (51.1) 22 (46.8) 4 (8.5) 15 (31.9) 3 (6.4) -	43 (46.2) 49 (52.7) 9 (9.7) 38 (40.4) 3 (3.2) 1 (1.1)
**Missing**	-	1 (2.1)	1 (1.1)
**Religion** Catholic Non catholic Evangelic Spiritualist Other No religion	20 (44.4) 25 (45.5) 16 (35.6) 1 (2.2) 4 (8.9) 4 (8.9)	19 (40.4) 28 (59.6) 18 (38.3) 3 (6.4) 2 (4.3) 5 (10.6)	39 (41.9) 53 (57.0) 34 (36.6) 4 (4.3) 6 (6.5) 9 (9.7)
**Missing**	1 (2.2)	-	1 (1.1)
**Marital status** Without partner Single Divorced Widow(er) With partner (married)	25 (54.3) 17 (37.0) 5 (10.9) 3 (6.5) 21 (45.7)	24 (51.1) 16 (34.0) 6 (12.8) 2 (4.3) 23 (48.9)	49 (52.7) 33 (35.5) 11 (11.8) 5 (5.4) 44 (47.3)
**Education** Incompl. High school or less Incompl. Primary school Compl. Primary school Incompl. High school Compl. High school or more Complete high school Incompl. university Compl. university	16 (34.8) 8 (17.4) 2 (4.3) 6 (13.0) 30 (65.2) 24 (52.2) 2 (4.3) 4 (8.7)	23 (48.9) 11 (23.4) 5 (10.6) 5 (10.6) 24 (51.1) 16 (34.0) 6 (12.8) 4 (8.5)	39 (41.9) 19 (20.4) 7 (7.5) 11 (11.8) 54 (58.1) 40 (43.0) 8 (8.6) 8 (8.6)
**Family income** No income / until 1 MW No income Until 1 MW Above 1 MW More than 1 to 2 More than 2 to 3 More than 3 to 5 More than 5 to 10	27 (58.7) 3 (6.5) 24 (52.2) 19 (41.3) 9 (19.6) 9 (19.6) 1 (2.2) -	24 (51.1) 2 (4.3) 22 (46.8) 23 (48.9) 15 (31.9) 3 (6.4) 2 (4.3) 3 (6.4)	51 (54.8) 5 (5.4) 46 (49.5) 42 (45.2) 24 (25.8) 12 (12.9) 3 (3.2) 3 (3.2)

Variables skin color (black / non-black), religion (catholic / nom catholic and no religion), marital status (with partner / without partner), education (incomplete high school or less / complete high school or more) and Family income (no income or to 1 minimum wage / above one minimum wage) were recategorized for statistical analysis.

^a^Mean (SD)CG, control group; Compl., complete; IG, intervention group; Incompl., incomplete; BMI, body mass index; MW, minimum wage.

Most participants had moderate and unintentional adherence according to MGLT. In most cases they reported to forget to take or neglect to take their medication. When comparing the baseline characteristics, CG had a higher proportion of intentional non-adherent participants when compared to the IG (Table 2).

**Table 2:** Distribution of adherence, clinical, and pharmacotherapeutic variables according to group allocation at baseline, Salvador, BA (2019–2021).

Variables	CG n = 46 (%)	IG n = 47 (%)	Total n (%)
Adherence levels Moderate Low	40 (87.0) 6 (13.0)	40 (85.1) 7 (14.9)	80 (86.0) 13 (14.0)
Adherence type Intentional Both Non-intentional	14 (30.4) 11 (23.9) 21 (45.7)	3 (6.4) 10 (21.3) 34 (72.3)	17 (18.3) 21 (22.6) 55 (59.1)
Pharmacotherapy			
Type of UC treatment Sulfasalazine Oral Mesalamine Rectal Mesalamine Azathioprine	11 (23.9) 30 (65.2) 27 (58.7) 2 (4.3)	13 (27.7) 26 (55.3) 36 (76.6) 4 (8.5)	24 (25.8) 56 (60.2) 63 (67.7) 6 (6.5)
N° of dosage units / day^a^	9.2 (4.0)	8.6 (3.6)	8.9 (3.8)
N° of medicines for other Diseases	1.5 (1.4)	1.6 (1.4)	1.5 (1.4)
Daily doses (mg) ^a^ Oral Mesalamine Rectal Mesalamine Sulfasalazine Azathioprine	3,480 (1,047) 944 (160) 3,682 (1,309) 100 (0)	3,123 (1,131) 924 (177) 2,731 (992) 175 (65)	3,314 (1091) 933 (169) 3,167 (1,222) 150 (63)
Disease activity (LI) Final score (0–21) Classification Remission Active disease Mild/moderate activity Severe colitis	5.9 (3.8) 18 (39.1) 28 (60.9) 22 (47.8) 6 (13.0)	5.1 (2.4) 19 (40.4) 28 (59.6) 27 (57.4) 1 (2.1)	5.5 (3.2) 37 (39.8) 56 (60.2) 49 (52.7) 7 (7.5)

^a^Considering enteral medication provided by public outpatient pharmacy for UC.

CG, control group; IG, intervention group; LI, Lichtiger Index.

To treat UC, most of the participants used rectal mesalamine and / or oral form. The average number of dosage units used per day for exclusive treatment of UC, including oral and rectal, was 8.9 (+ 3.8) per participant. The average daily doses of drugs used to treat UC are available in Table 2. The average number of drugs used for other clinical conditions was 1.5 (+ 1.4) drugs per participant. Regarding the severity of UC, the mean LI score was 5.5 (+ 3.2), which corresponds to a mild or moderate activity (Table 2).

Comparative groups at baseline were homogeneous regarding all eight QoL domains.

### 3.2. Impact of interventions on primary outcome

It was observed that there were no significant differences in adherence (classified according to MGLT), when compared by allocation groups. The descriptive data on adherence before and after interventions are shown in Table 3 and the comparative analysis is shown in Table 4.

**Table 3:** Descriptive data of primary outcome (adherence according to MGLT) before and after intervention program according to group allocation, Salvador, BA (2019–2021).

Adherence before interventions	Adherence after interventions			
	High	Moderate	Low	TOTAL
Intervention Group (n = 43)				
Moderate	13 (36.1)	22 (61.1)	1 (2.8)	36 (100.0)
Low	4 (57.1)	2 (28.6)	1 (14.3)	7 (100.0)
TOTAL	17 (39.5)	24 (55.8)	2 (4.7)	43 (100.0)
Control Group (n = 40)				
Moderate	18 (51.4)	17 (48.6)	-	35 (100.0)
Low	-	3 (60.0)	2 (40.0)	5 (100.0)
TOTAL	18 (45.0)	20 (50.0)	2 (5.0)	40 (100.0)

**Table 4:** Comparison of primary outcome (adherence according to MGLT) 6 months after the intervention program according to group allocation, Salvador, BA (2019–2021).

	Total N	Maximum adherence ^a^ N (%)	RR (CI95%)	*p*	Improved adherence ^b^ N (%)	RR (CI95%)	*p*
IG	43	17 (39.5)	0.879 (0.53–1.45)	0.291	19 (44.2)	0.842 (0.54–1.32)	0.766
CG	40	18 (45.0)	1.0		21 (52.5)	1.0	

^a^Proportion of participants who improved to maximum level of adherence after interventions.

^b^Proportion of participants who improved to a moderate or maximum level of adherence after interventions.

CI, confidence interval; CG, control group; IG, intervention group; RR, relative risk.

Given that the type of non-adherence (unintentional / both / intentional) was different at the baseline between the IG and CG, the effect of this condition as a confounder was assessed. After adjustment, the odds ratio of 0.64 (95% CI: 0.21–1.93; p = 0.423) and OR 0.44 (95% CI: 0.11–1.74; p = 0.245) was observed for the outcome of maximum adherence. Similarly, an adjusted OR of 0.50 (95% CI: 0.16–1.54; p = 0.227) and an OR 0.88 (95% CI: 0.23–3.34; p = 0.845) were observed for those who improved adherence to moderate or maximum adherence, indicating absence of confounding effects by type of non-adherence. It is noteworthy that even if all those lost to follow-up from the IG were adherent (4 participants), still the results would not have changed.

### 3.3. Impact of interventions on secondary outcome

The comparative analysis of the differences in mean scores at Visit 2 and 1, using paired and independent Student’s t-test, revealed that there was a significant improvement in three QoL domains for the IG compared to the CG: “Pain” and “Vitality” and an improvement trend for the “Emotional aspects” domain (Table 5).

**Table 5:** Comparison of secondary outcome (quality of life) before and after the intervention program between allocation groups, Salvador, BA (2019–2021).

	Intervention Group (n = 43)				Control Group (n = 40)				
	Before	After	Mean	*p* ^a^	Before	After	Mean	*p* ^a^	*p* ^b^
QoL domains	Mean (SD)	Mean (SD)	of differences (SD)		Mean (SD)	Mean (SD)	of differences (SD)		Differences (IG *vs* CG)
Functional capacity	72.3 (27.2)	76.2 (29.0)	3.8 (16.9)	0.145	72.4 (25.0)	72.5 (26.3)	0.1 (19.6)	0.968	0.358
Physical aspects	50.6 (41.0)	59.4 (39.2)	8.8 (43.9)	0.194	56.9 (35.3)	49.4 (39.4)	-7.5 (45.7)	0.306	0.101
Pain	53.0 (25.4)	60.2 (27.6)	7.2 (29.8)	0.123	58.5 (23.7)	47.5 (23.0)	-11.0 (26.0)	0.011	0.004
General health state	53.6 (21.7)	54.0 (22.6)	0.4 (15.9)	0.883	56.8 (21.5)	56.0 (20.7)	-0.8 (16.7)	0.770	0.752
Vitality	49.9 (23.2)	54.2 (24.5)	4.3 (19.0)	0.147	56.0 (22.2)	49.4 (18.6)	-6.6 (20.1)	0.043	0.013
Social aspects	67.2 (28.6)	69.8 (30.8)	2.6 (32.4)	0.596	72.4 (22.0)	68.2 (28.7)	-4.2 (24.7)	0.292	0.287
Emotional aspects	51.9 (38.7)	62.0 (42.8)	10.1 (53.3)	0.222	66.6 (40.0)	50.0 (41.3)	-16.6 (54.5)	0.061	0.027
Mental health	60.6 (22.1)	59.3 (22.4)	-1.3 (21.5)	0.694	62.7 (22.0)	59.4 (23.8)	-3.3 (25.4)	0.416	0.700

^a^Paired Student’s t-test.

^b^Independent Student’s t-test.

CG, control group; IG, intervention group; QoL, quality of life.

To confirm possible confounders for the previously highlighted result, the median scores at baseline for "Pain", "Vitality" and "Emotional aspects" domains were analyzed according to age, sex, marital status, education, income, and type of non-adherence. Marital status, clinical status, education, and income were identified as potential confounders (Table 6).

**Table 6:** Crude and adjusted β coefficient (95%CI) of final and initial quality of life scores differences to compare control group (n = 40) and intervention group (n = 43), Salvador, BA (2019–2021).

Quality of life^a^ (domains)	Model without adjustments β (CI95%)	Model 1^b^ β (CI95%)	Model 2 β (CI95%)	Model 3^e^ β (CI95%)
Pain	18.125** (5.861 a 30.389)	17.963** (5.621 a 30.306)	17.845^c^** (5.811 a 29.878)	18.352** (6.121 a 30.582)
Vitality	10.904* (2.369 a 19.439)	10.568* (2.078 a 19.059)	-	-
Emotional aspects	26.710* (3.162 a 50.257)	27.038* (3.343 a 50.733)	24.907^d^* (1.104 a 48.710)	-

^a^Mean of quality of life domain differences between 6 and 0 months were calculated for each domain.

^b^Adjusted for disease activity.

^c^Adjusted for disease activity and marital status.

^d^Adjusted for disease activity, income and education.

^e^Adjusted for disease activity, marital status and sex.

*p<0.05

**p<0.01.

In the multivariate analysis, it was observed that interventions remained effective as promoters of better QoL scores for the “Pain”, “Vitality”, and “Emotional Aspects”, when adjusted (Table 6).

## 4. Discussion

The intervention program was not able to produce significant differences for the primary outcome (medication adherence), but was able to improve three QoL domains.

The need to include non-adherent patients in clinical trials had previously been signaled as a strategy to improve the quality of published studies [13]. A later published analysis found that out of 190 clinical trials, only six had clearly adopted non-adherent patients as an inclusion criterion, with none of these targeting IBD patients [32]. It was not possible to directly compare the results with published articles, since the present study included only patients classified as non-adherent at baseline, being a strong point of the research protocol.

Most participants were women, with an average age of 47 years, black or mixed race, residing in urban area. It seems that in Latin America there is a predominance of women and residents in urban areas [33], as opposed to international studies that do not point out differences in the sex ratio for UC patients [34, 35].

As for the clinical status for UC, most of them had active disease at baseline, comparable to the proportion of patients with moderate to severe UC in Brazil [33]. Other studies in the country have found inverse results, with a higher proportion of patients with disease in remission, differing from the results presented here in [31, 36]. It is noteworthy that the participants were assisted at a pharmaceutical service, while in other studies in Brazil, participants were interviewed during medical care assistance. In previous studies, mild disease activity was associated with increased risk of medication adherence [37, 38].

Most participants used oral and/or rectal amino salicylates. Findings converge with previously published results, where the largest proportion of patients were using salicylates for UC [33, 39, 40].

It is necessary to emphasize that rectal medication use for IBD treatment has been considered a risk factor for low adherence when compared to the oral route. This is possibly due to the discomfort related to administration, difficulty in use during working hours or on trips, as well as the lack of adherence in these cases has been associated with intentional behavior [41–44]. This may have influenced unfavorably to the research intervention program, since most participants were using the rectal route.

At baseline, significant differences were observed in the distribution of types of non-adherence between groups. Studies with similar objectives that used MGLT, with patients diagnosed with UC, did not show baseline distribution of this variable, therefore, we could not compare how this variable behaved in other studies [45, 46]. The theoretical framework of the Medication Adherence Model considers that intentional and unintended aspects cannot be seen in a fragmented way, but interrelated [47]. Therefore, it seems that the imbalance in the type of non-adherence in the groups did not interfere in the results, given that the interventions proposed in the study would have possible repercussions in sub-items for both types of intentionality.

The intervention program proposed by this research is considered to be multicomponent, as it contains multiple strategies, educational, behavioral and motivational [48]. Multicomponent type interventions are reported to be more effective, as they address possible different causes of non-adherence to drug treatment [22, 49]. However, the results did not demonstrate that exposure to the intervention program had any impact on improving adherence, even after adjusting for confusion. The results pointed to a high number of daily dosage units used by the studied sample, and previous studies indicate an inverse relationship between the number of doses of the drugs in use and adherence [2], which may have reduced the effectiveness of interventions performed for adherence outcome.

The multicomponent approach can be complex, as it involves different combinations of interventions with regard to type, frequency, intensity and quality [48]. In addition, the measurement of adherence depends on the method chosen, therefore, these points make it difficult to directly compare results between studies. There are few data published with similar objectives that evaluated multicomponent interventions for UC patients [49], and results are conflicting [45, 50, 51].

In the present study, educational measures were carried out in a single moment, while motivational and reminder messages were repeated. It is possible that the most immediate result of these interventions was not captured due to the time elapsed until data collection at visit 2 (after 6 months), therefore, an increase on the frequency of educational intervention and instruments application to measure adherence would be desirable. In a meta-analysis it was found that the longer the follow-up time, the smaller the differences for the outcome of adherence between comparative groups [52]. Another study in Denmark and Ireland, with multicomponent type interventions, found no result in improving adherence after twelve months in patients with UC, however, it demonstrated an impact at four weeks [50]. These results can signal the effect durability of the interventions, as well as to understand that adherence is a dynamic phenomenon.

It is possible that the intervention program of this research did not have a greater impact because it did not include changes in the interaction routine between participants and health professionals. Multiple interventions through a virtual platform have already been proposed, in which, in addition to educational approach and reminders, the data entered by patients were analyzed using a pre-defined automatic protocol, and if necessary, the assistance team was activated for appropriate measures. Del Hoyo *et al*. [53] found significant improvement in adherence after 24 weeks. The program of interventions in this work suggests, for analysis in future research, the association of other formats of patient care with a multidisciplinary team, including pharmacists, in order to enhance their effects [54, 55].

As for QoL, in this research there was an improvement in the scores of the domains “Pain” and “Vitality” in the IG when compared to the CG, a result maintained after adjustment for confounders. Other studies have already found the impact of multicomponent interventions with an improvement in QoL. Although Cross *et al*. [45] did not observe an improvement in the outcome of adherence, they found an improvement in QoL, measured using a validated instrument specific to IBD (Inflammatory Bowel Disease Questionnaire ‐ IBDQ), of the exposed group, when compared to the unexposed after adjustment for knowledge baseline disease. Elkjaer *et al*. [50] used the SF-36 instruments and the short version of the IBDQ, and found results similar to those obtained in the present study, for “Vitality” and, additionally, they found improvement for “Health in general”, "Emotional function" and "Social aspects", while in the present study there was an additional improvement for "Pain". We emphasize that the SF-36 pain domain evaluates pain from a subjective perspective, based on the individual’s self-report. The questionnaire translates the individual perception of pain and its impact on the subject’s functioning and well-being. Therefore, it is possible that the intervention program of the present study had an impact on pain perception, while it was not able to change medication adherence behaviors.

According to observed results in the present research, multicomponent interventions may contribute to improve QoL for UC patients, despite no significant impact for medication adherence having been found. Other clinical trials aiming to improve adherence to chronic diseases have shown improvement in QoL [13] and multicomponent approaches seem to trigger better outcomes [56]. Interventions improving IBD population QoL are important not only for medical reasons, but for their social implications and the demand for health resources [57, 58].

Some limitations of the present study may have interfered with the observed results. The sample size was calculated to identify differences of 30% in the primary outcome, therefore, it is possible that statistical differences in adherence between the comparative groups were not observed due to insufficient power. It is suggested that in future designs, differences of lesser magnitude of the outcome should be considered for the sample calculation. Another point was the impossibility of blinding the participants regarding the allocation, exposing the results to the risk of performance bias, eventually improving the provision of usual attention perceived by the CG participants, which may have attenuated the differences between the groups [59]. On the other hand, the interviewer at visit 2 did not have access to the allocation, which protected the data collection and results calculation. It is important to emphasize that the outcomes of this research are based on self-reported data, which makes them susceptible to the ability to remember and depends on the respondent’s contribution. Disease specific MGLT and QoL scores were not used due to limited financial resources.

We must discuss the atypical moment experienced during the execution of the research after declaring the state of emergency due to the new coronavirus, which may have affected participants’ QoL and adherence to treatment. A recent study identified that stress due to the pandemic was reported by 22% of IBD patients as high and 38% as moderate. In addition, 62% of the patients had concern about medications increasing the risk of developing COVID-19. Additionally, 14% of patients considered discontinuing, while 11% effectively discontinued treatment because of pandemic [60], results confirmed by patients with rheumatic diseases [61]. The interest for research on the impact of COVID-19 on adherence to treatment has been pointed out [62], but results of clinical trials with this purpose have not yet appeared in the scientific literature. Recent studies pointed conflicting results regarding COVID-19 pandemic impact on medication adherence [60, 63], signaling an important gap to be addressed, both for IBD and for other chronic diseases.

## 5. Conclusion

Exposure to the proposed intervention program was not able to produce a significant difference between comparative groups in medication adherence for non-adherent patients with UC. We suggest that in future studies, outcome differences of lesser magnitude for sample calculation, shorter intervals for measuring adherence using more than one method, higher frequency of educational interventions and inclusion of new forms of interaction between patient and care team should be considered.

In this research, the intervention program demonstrated a significant improvement for QoL, specifically for the domains of "Pain", "Vitality" and "Emotional aspects". This result is extremely important for UC patients, as it is a chronic disease with a recognized negative impact on QoL.

## Supporting information

S1 File Protocol. (DOCX)

S2 File CONSORT checklist. (DOC)

S3 File CONSERVE checklist. (DOCX)

S4 File Minimal data set. (XLSX)
