# Supplementary material for: Impact of an intervention program on drug adherence in patients with ulcerative colitis: Randomized clinical trial
Source: PLoS One. 2023 Dec 27;18(12):e0295832. doi: 10.1371/journal.pone.0295832 (PMC10752503; doi:10.1371/journal.pone.0295832)
Supplement: S1 File — (DOCX) [file pone.0295832.s001.docx]

# Impact of an Intervention Program on Drug Non-Adherence in Ulcerative Colitis Patients: Randomized Clinical Trial

GENOILE OLIVEIRA SANTANA SILVA RAQUEL ROCHA DOS SANTOS

NEY BOA-SORTE MILA PALMA PACHECO

Salvador November / 2018

#### ABSTRACT

Some authors point low adherence to drug treatment as a public health problem with a variable magnitude of 15% to 93% for patients with chronic diseases, being characterized as an "invisible epidemic". In Brazil, few clinical trials were identified evaluating interventions with primary outcome on non-adherence, with most of the participants being carriers of cardiovascular diseases and none of those with inflammatory bowel diseases (IBD). There is a gap in knowledge production of interventions which may impact the non-adherence of patients with IBD, evaluated through studies with randomized clinical trial design, characterizing this study as innovative. IBD are part of the Clinical Protocols and Therapeutic Guidelines of the Ministry of Health with wide coverage of oral and parenteral drug treatment. For this reason, it is of fundamental importance to evaluate and implement measures with the aim of increasing adherence to the prescribed treatment. The present study has as general objective to evaluate the impact of an intervention program in non-adherence to drug treatment in patients with Ulcerative Colitis (UC). This is a prospective longitudinal experimental study with a pragmatic randomized clinical trial design, scheduled to begin in March 2019. Semi-structured interview technique will be employed using the Brasil SF-36 and the Morisky-Green-Levine test, in addition to questionnaires prepared specifically for this study for data collection and secondary data obtained from participants' records, when necessary. The data will be inserted in a database built with the statistical package SPSS, version 21.0. Participants of the Control Group (CG) will not receive the interventions, while those of the Intervention Group (IG) will receive the intervention program individually in a private setting. The program will consist of educational interventions and behavioral interventions applied for a period of 6 months. Socio-demographic, economic, clinical, pharmacotherapeutic, non-adherence and quality of life variables will be analyzed. It will be considered a bilateral (1-alpha) significance level of 95%, power (1-beta) with 80% probability of detection, sample size ratio between exposed / unexposed of 1, percentage of unexposed positive 50% and percentage of exposed positive 80%, Odds Ratio: 4, risk ratio / prevalence of 1.6 and risk / prevalence difference of 30, calculation generated through OpenEpi, version 3.01, was calculated 45 participants in each group (GC and GI) for RCU patients with a total

sample size of 90. Quantitative variables will be described as means (standard deviation) or medians (interquartile range), while qualitative variables will be described with absolute and percentage frequency measures. To compare the exposed (GI) and non-exposed (CG) groups in relation to the qualitative variables, the chi-square test or Fischer's exact test, when recommended, will be used. The comparison of the quantitative variables between the exposure groups will be performed by applying the Student t test for unpaired samples or, if indicated, the non-parametric Mann-Whitney test. The occurrence (incidence) of the study outcomes (non-adherence) will be calculated by the ratio of the total number of participants non-adhering to the treatment and the total number of participants in the group, multiplied by 100, calculated separately for the intervention and comparison groups. The relative risk (RR) with the respective 95% confidence interval (95% CI) will be used as a measure of effectiveness, obtained by the ratio between the incidence of the intervention group and the incidence of the comparative group. For adjustments for potential confounders, Poisson regression with robust variance will be used to estimate RR adjusted with their respective 95% CI. Associations with p values less than 0.05 will be considered significant.

**Key-words**: medication adherence, randomized controlled trial, ulcerative colitis.

#### INTRODUCTION

Some authors point to low adherence to drug treatment as a public health problem with a magnitude varying from 15 to 93% for patients with chronic diseases, with an estimated average of 50%, depending on the method used, characterized as an "invisible epidemic". The lack of adherence generates direct and indirect costs, in addition to clinical, social and environmental repercussions, and it is therefore important not only to identify its magnitude, but also to plan and implement solutions to effectively address this issue[2].

Faced with this reality, global strategies have been presented as promising in the engagement against non-adherence. In 2007, the Brazilian Ministry of Health published the "Guidelines for the Strengthening of Care Actions for People Living with HIV/AIDS"[3] and in 2016 entitled "Synthesis of Evidence for Health Policies: Adherence to drug treatment of patients with chronic diseases"[4]. These texts present the global panorama of the lack of adhesion and possibilities of confrontation, based on international studies.

Inflammatory bowel diseases are part of the Clinical Protocols and Therapeutic Guidelines of the Ministry of Health [5] with wide coverage of treatment with the provision of oral and parenteral drugs. For this reason, the evaluation and implementation of measures to increase adherence to prescribed treatment is of fundamental importance.

#### AIMS

**GENERAL AIM**

To evaluate the impact of an intervention program on the rate of non-adherence to drug treatment in patients with Ulcerative Colitis (UC).

#### SPECIFIC AIMS

1. To assess possible factors associated with the impact of an intervention program on non-adherence to drug treatment in patients with UC;
2. To evaluate the impact of an intervention program on the quality of life of patients with UC.

#### PROPOSED METHODOLOGY

**Study Location**

The study will be carried out at the Integrated Pharmacy of Medicines in Specialized Care (FIMAE) in partnership with the IBD outpatient clinics of the Roberto Santos General Hospital (HGRS) and the Professor Edgard Santos University Hospital of UFBA (HUPES), which are references in Inflammatory Diseases. Intestinal (IBD). FIMAE is a public unit of the Health Department of the State of Bahia (SESAB) responsible for dispensing medication to patients with IBD.

#### Study Design

This is a prospective longitudinal experimental study with a pragmatic randomized clinical trial design, in which attention will be paid to the preservation of routine care and service conditions, to favor the application of the study results to the operational conditions of the service in question. The study is scheduled to start in March 2019 with a duration of 36 months.

#### Target Audience

The target audience will be patients diagnosed with UC in the State of Bahia treated by the Unified Health System (SUS) at FIMAE.

#### Ethical Aspectcs

The study will be submitted to the Research Ethics Committee of the Universidade do Estado da Bahia (CEP/UNEB) through Plataforma Brasil for analysis. After approval of the project by the CEP, the Free and Informed Consent Term (ICF) (Appendix 1) will be presented and signed by the patients before entering the research for a subsequent interview, application of the research instruments, as well as a review of medical records and interventions.

In order to guarantee the safety of the information collected, the computers will be used exclusively for research and will be configured to lock with a password that is restricted to designated researchers. All physical documentation will be kept in its own locker with a restricted access key, also to protect the integrity of the information [6].

#### Inclusion Criteria

Patients diagnosed with UC who, during the capture stage, meet all the criteria listed below will be included in the research: they have at least 3 months of active registration at FIMAE; prescribed with at least 01 (one) drug to treat UC; aged 18 years or over; able to respond to the research instruments; who agree to sign the TCLE; that they are assisted by the IBD outpatient clinics at the HGRS or HUPES; who have a cell phone.

#### Exclusion Criteria

Patients who do not meet the research criteria will be excluded, as well as patients who are classified as adherent during the recruitment stage.

#### Participants’ Allocation

After signing the informed consent, the participants will be submitted to the research protocol (Appendix 2). They will be allocated to an Intervention Group (IG) or Control Group (CG) using a randomization technique using a previously generated list of random numbers, before allocation. Allocation confidentiality will be preserved, as the researchers who will carry out the intervention and measure the outcomes will be different, therefore, those who will carry out the outcome assessment will be “blind” and will not know in which group each participant was allocated.

#### Data Collection

Data collection will be carried out by a team of scientific initiation scholarship holders, duly trained and qualified, under the guidance of the other researchers, including the course of Good Clinical Practices through a web platform (https://gcp.nidatraining.org/). We will use the semi-structured interview technique with questionnaires designed specifically for this study for initial data collection, prior to the proposed intervention program (Appendix 3), and at the end after 6 months of the

intervention program (Appendix 4). Validated instruments for the Brazilian reality will be used, such as the Brasil SF-36[7], which captures the participants' quality of life and the Morisky-Green-Levine Test (MGLT)[8] to measure the degree of non-adherence, as well as the Lichtiger Index[9] to determine the clinical activity of UC. We will also collect secondary data from the participants' medical records, when necessary. A form will be used to record the interventions carried out that will make up the intervention program (Appendix 5). The data will be entered in a database built with the SPSS® statistical package, version 21.0.

#### Blinding

Participants will be identified with a code throughout the study, their names being omitted. The table with names and codes will be available only to those responsible for implementing the interventions (two non-blind researchers). Two other researchers will be responsible for collecting and entering the database in duplicate, to reduce the chances of errors at this stage. Two blind researchers different from the previous ones will do the analysis of the database in duplicate.

#### Intervention Program

Participants in the CG will not receive the interventions, while those in the IG will receive the intervention program individually in a private environment. The intervention program will consist of educational interventions and behavioral interventions, following previously established frequencies, as explained below.

Educational Interventions:

- A video lasting about 5 minutes will be played, presenting basic content about IBD and the importance of adherence to prescribed drug treatment, prepared by the research team according to the script established in Appendix 6 and text in Appendix 7. The video is available at the following link: link: https://youtu.be/vcvm9DXAXNg. This intervention will be carried out at Time 0;
- A printed educational leaflet (Appendix 8) will be delivered, presenting basic contents about UC and the importance of adherence to prescribed drug treatment, prepared by the research team based on available scientific evidence,

according to the script established in Appendix 9. The subject will have 10 minutes for silent reading, and at the end, the team will be available to answer the questions of the participants. This intervention will be carried out at Time 0;

- will be oriented on the drugs prescribed for UC and available for dispensing at FIMAE (Appendix 10) according to the list of clinical protocols[5]. Verbal guidelines will follow the script described in Appendix 11, using as reference the drug monographs available in the Micromedex® database[10]. This intervention will be carried out: at Time 0, or when there is a change in medication during the study period, or when the participant has doubts about medication;

Behavioral Interventions:

- a therapeutic regimen will be prepared according to the model shown in Appendix 12. Participants who demonstrate difficulties in understanding the written guidelines will additionally receive a Pharmaceutical Guidance Table[11] (Anexx A), ), in order to facilitate the understanding of the times and quantities of each prescribed drug. This intervention will be carried out: at Time 0; or when there is a change of medication during the study period; or when the participant has doubts about the therapeutic regimen;
- Short Message Service (SMS) messages will be forwarded to the cell phones registered by the participants:
  - reminder messages of the service date for acquiring medication at FIMAE (Appendix 13). This intervention will be carried out at least 2 days before the return date and 2 hours before the scheduled time;
  - motivational messages with the contents exemplified in Appendix 14. This intervention will be carried out once a week.

#### Data Categorization

Socio-demographic and economic variables will be analyzed:

- - sex, age, ethnicity/color, origin, marital status, monthly income, education.

Pharmacotherapeutic variables will be analyzed:

- - prescription drugs classified according to the Anatomical Therapeutic Chemical Classification (ATC)[12] for UC and comorbidities, at levels 3 and

5. This variable will be collected at Time 0 and if there is a change in treatment;

- - therapeutic regimens for UC. This variable will be collected at Time 0 and if there are changes in treatment;
  - self-reported drug non-adherence for drugs being treated for UC, according to MGLT[8]. This variable will be collected at Time 0 and 6 months;
  - number of educational interventions of the “Drug Guidance” type: frequency of guidance per drug per patient carried out during the intervention program;
  - number of behavioral interventions of the “Therapeutic Scheme” type: frequency of therapeutic schemes per patient performed during the intervention program;
  - number of behavioral interventions of the “Pharmaceutical Guidance Table” type: frequency of the table per therapeutic regimen per patient, performed during the intervention program;
  - number of behavioral interventions of the type “SMS Reminder Type”: frequency of SMS reminder type per patient, performed during the duration of the intervention program;

Clinical variables will be analyzed:

- - disease activity - Lichtiger index[9], which establishes the clinical status of UC. This variable will be collected at Time 0 and 6 months;

Subjective variables reported by the patient will be analyzed (Patient Reported Outcomes - PRO):

- - quality of life. This variable will be collected at Time 0 and 6 months using the Brazil SF-36 questionnaire[53];

#### Sample Calculation

We will consider a bilateral significance level (1-alpha) of 95%, power (1-beta) with 80% probability of detection, sample size ratio between exposed/unexposed of 1, percentage of positive unexposed of 50%[13] and percentage of positive exposure of 80%, Odds Ratio: 4, risk/prevalence ratio of 1.6 and risk/prevalence difference of 30, the calculation generated through OpenEpi, version 3.01, calculated 45 participants in each group (CG and IG) for UC patients, with a sample size of 90.

#### Statiscal Analysis

Quantitative variables (age, disease duration, UC severity score, for example) will be described as means (standard deviation) or medians (interquartile range), while qualitative variables (gender, marital status, education, for example) will be described with absolute and percentage frequency measures. To compare exposed (GI) and non- exposed (CG) groups in relation to qualitative variables, the chi-square test or Fisher's exact test will be used, when recommended. The comparison of quantitative variables between exposure groups will be performed by applying Student's t test for unpaired samples or, if indicated, the nonparametric Mann-Whitney test.

The occurrence (incidence) of the study outcomes (non-adherence) will be performed by the ratio between the total number of participants who did not adhere to the treatment and the total number of participants in the group, multiplied by 100, calculated separately for the intervention and comparative groups. The relative risk (RR) with the respective 95% confidence interval (95%CI) will be used as a measure of efficacy, obtained by the ratio between the incidence of the intervention group and the incidence of the comparative group. For adjustments for potential confounders, Poisson regression with robust variance will be used to estimate adjusted RR with the respective 95%CI. Associations with p values lower than 0.05 will be considered significant.

#### REFERENCES

1. Jakovljevic M. Non-adherence to medication: a challenge for person-centred pharmacotherapy to resolve the problem. *Psychiatr Danub* 2014; **26 Suppl 2**: 358-363 [PMID: 25433315]
2. Adherence to long-term therapies: evidence for action. Switzerland: World Health Organization, 2003
3. Diretrizes para o fortalecimento das ações de adesão ao tratamento para pessoas que vivem com HIV e AIDS. Brazilian Ministry of Health, 2007: 32
4. Síntese de evidências para políticas de saúde: adesão ao tratamento medicamentoso por pacientes portadores de doenças crônicas. Brazilian Ministry of Health, 2016: 54
5. Portaria SAS/MS n 861. Brasília: Ministério da Saúde, 2002
6. Mocciaro F, Di Mitri R, Russo G, Leone S, Quercia V. Motivational interviewing in inflammatory bowel disease patients: a useful tool for outpatient counselling. *Dig Liver Dis* 2014; **46**(10): 893-897 [PMID: 25085685 DOI: 10.1016/j.dld.2014.07.009]
7. Ciconelli RM. Tradução para o português e validação do questionário genérico de avaliação de qualidade de vida "Medical outcomes study 36-item short-form health survey (SF- 36)". Medicine School. São Paulo: Escola Paulista de Medicina, 1997: 120
8. Ben ÂJ. Confiabilidade e análise de desempenho de dois questionários de avaliação da adesão ao tratamento anti-hipertensivo: teste de Morisky-Green e Brief Medication Questionnaire. Medicine School. Porto Alegre: Universidade Federal do Rio Grande do Sul, 2011: 106
9. Lichtiger S, Present DH, Kornbluth A, Gelernt I, Bauer J, Galler G, Michelassi F, Hanauer S. Cyclosporine in severe ulcerative colitis refractory to steroid therapy. *N Engl J Med* 1994; **330**(26): 1841-1845 [PMID: 8196726 DOI: 10.1056/NEJM199406303302601]
10. Micromedex. Truven Health Analytics Inc., 2018
11. Cani CG. Impacto da atenção farmacêutica no cuidado de pacientes portadores de diabete melito tipo 2 atendidos em hospital de nível terciário de atenção. Medicine School. São Paulo: Universidade de São Paulo, 2011: 123
12. ATC classification index with DDDs. Oslo, Norway: WHO Collaborating Centre for Drug Statistics Methodology, 2017
13. Dewulf NLS. Adesão ao tratamento medicamentoso de pacientes com doenças inflamatórias intestinais acompanhados no ambulatório de um hospital universitário. *Arq Gastroenterol* 2007; **44**(4): 8

#### Appendice 1 – Informed Consent Form

**UNIVERSIDADE DO ESTADO DA BAHIA DEPARTAMENTO DE CIÊNCIAS DA VIDA CAMPUS I COLEGIADO DE FARMÁCIA / PPGFARMA**

#### - Identification data

Participant's name:

Identity number:

Gender:

Birth date:

Address:

Telephone:

#### - Research data:

1. Research title: "Impact of an intervention program on non-adherence to medication in patients with ulcerative colitis: a randomized clinical trial"
2. Responsible researcher: Genoile Oliveira Santana Silva Position/function: Coordinator

#### - Researcher explanations to the research participant:

You are being invited to participate in the research: "Impact of an intervention program on non-adherence to medication in patients with ulcerative colitis: a randomized clinical trial", by the researcher Genoile Oliveira Santana Silva, a professor at the University of the State of Bahia whose objective is to evaluate the impact of an intervention program on non-adherence to medication in patients with ulcerative colitis. The accomplishment of this research treats or can bring benefits to the improvement in the quality of life. If you accept, an interview will be carried out to answer questionnaires by students of undergraduate courses at UNEB, designated by the coordinator.

Questions will be asked about personal data (age, address, telephone, where I was born and where I live) and questions about the disease, medication and quality of life. The interviews will be documented and kept in a safe place. Information will also be collected in the medical records on the amount of drugs received for treatment for rectocolitis. Participants in this research will be drawn to receive or not interventions such as guidelines, videos and messages, and they can be contacted by the team through the telephone numbers provided. Due to the collection of information, you may feel uncomfortable/embarrassed.

Your participation is voluntary and there will be no expense or remuneration resulting from it. You will be reimbursed when there are travel costs to participate in the research activities. We guarantee that your identity will be treated safely, and therefore, you will not be identified. If you wish, you may, at any time, withdraw from participating and withdraw your authorization. Your refusal will not affect your relationship with the researcher or the institution.

Any doubts that you present will be clarified by the researcher and, if you wish, you can also contact the Ethics Committee of the Universidade do Estado da Bahia. I also clarify that according to Brazilian law you are entitled to compensation if you are harmed by this research. You will receive a copy of this term containing the contact of the researchers, who will be able to answer your questions about the project and your participation, now or at any time.

#### - INFORMATION ON NAMES, ADDRESSES AND PHONE PHONE OF THE RESPONSIBLE FOR MONITORING THE RESEARCH, FOR CONTACT IN CASE OF DOUBTS:

**RESPONSIBLE RESEARCHER: Genoile Oliveira Santana Silva**

Address: Rua Silveira Martins, 2555, Cabula, Phone: (71) 99957-2121. Email: [genoile@uol.com.br](mailto:genoile@uol.com.br)

#### Research Ethics Committee - CEP/UNEB

Address: Avenida Engenheiro Oscar Pontes s/n, former Petrobras building 2nd floor, room 23, Água de Meninos, Salvador-BA, CEP: 40.460-120.

Phone: (71) 3312-3420, (71) 3312-5057, (71) 3312-3393 ext. 250, E-mail:

[cepuneb@uneb.br](mailto:cepuneb@uneb.br)

#### National Research Ethics Commission - CONEP

Address: SRTV 701, Via W 5 Norte, lot D - Building PO 6700, 3rd floor - Asa Norte. CEP: 70719-040, Brasília-DF

#### - POST-INFORMED CONSENT

After being duly informed by the researcher about the objectives and benefits of the research and risks of my participation in the research "Impact of an intervention program on non-adherence to medication in patients with ulcerative colitis: a randomized clinical trial" and having understood what has been explained to me, I agree to participate of my own free will, as a volunteer I consent to the results obtained being presented and published in scientific events and articles as long as my identification is not carried out and I will sign this document in two copies, one for the researcher and another to me.

Date: / /

|  |  |  |
| --- | --- | --- |
| Participant’s signature |  | Researcher’s signature |

**Appendice 2 – Research Protocol Flowchart**


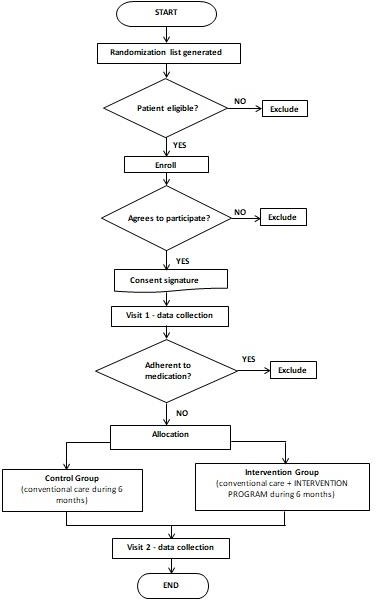


#### Appendice 3 – Survey Questionnaire for initial data collection

- 1. Participant identification code: | | | | |
  2. Date (time 0): / /
  3. Interviewer:

**SOCIO-DEMOGRAPHIC DATA**

- 1. Procedence: (1) Countryside (2) Urban (9) Not applicable (NA)
  2. Sexo: (1) masculino (2) feminino (9) NA
  3. Birth date: / /
  4. Age (Years): ( )
  5. Ethnicity/collor (self-declared): (1) black (2) white (3) mixed race (4) yellow (5) indigenous

(6) no declaration. (9) NA

- 1. Children: (1)one (2)two (3)three or more (9)NA
  2. Religion: (1)Catholic (2)Evangelic (3)Spiritualist (4) No religion (9)NA
  3. Marital status: (1) Single (2) Married (3) Divorced (4) Widow(er) (9) NA
  4. Education: (1) Incomplete primary school (2) Complete primary school (3) Incomplete highschool

(4) Complete highschool (5) Incomplete university (6) Complete university (9) NA.

- 1. Family income (minimum wage): (1) no income, up to 1 (2) more than 1, up to 2 (3) more than 2,

up to 3 (4) more than 3, up to 5 (5) more than 5, up to 10 (6) more than 10, up to 20 (7) more

than 20 (8) NA

**QUALITY OF LIFE DATA (BRASIL SF 36)**

**Instructions**: this survey asks you about your health. This information will keep us informed of how you feel and how well you are able to carry out your activities of daily living. Answer each question by marking the answer as indicated. If you are unsure how to respond, please try to answer as best you can.


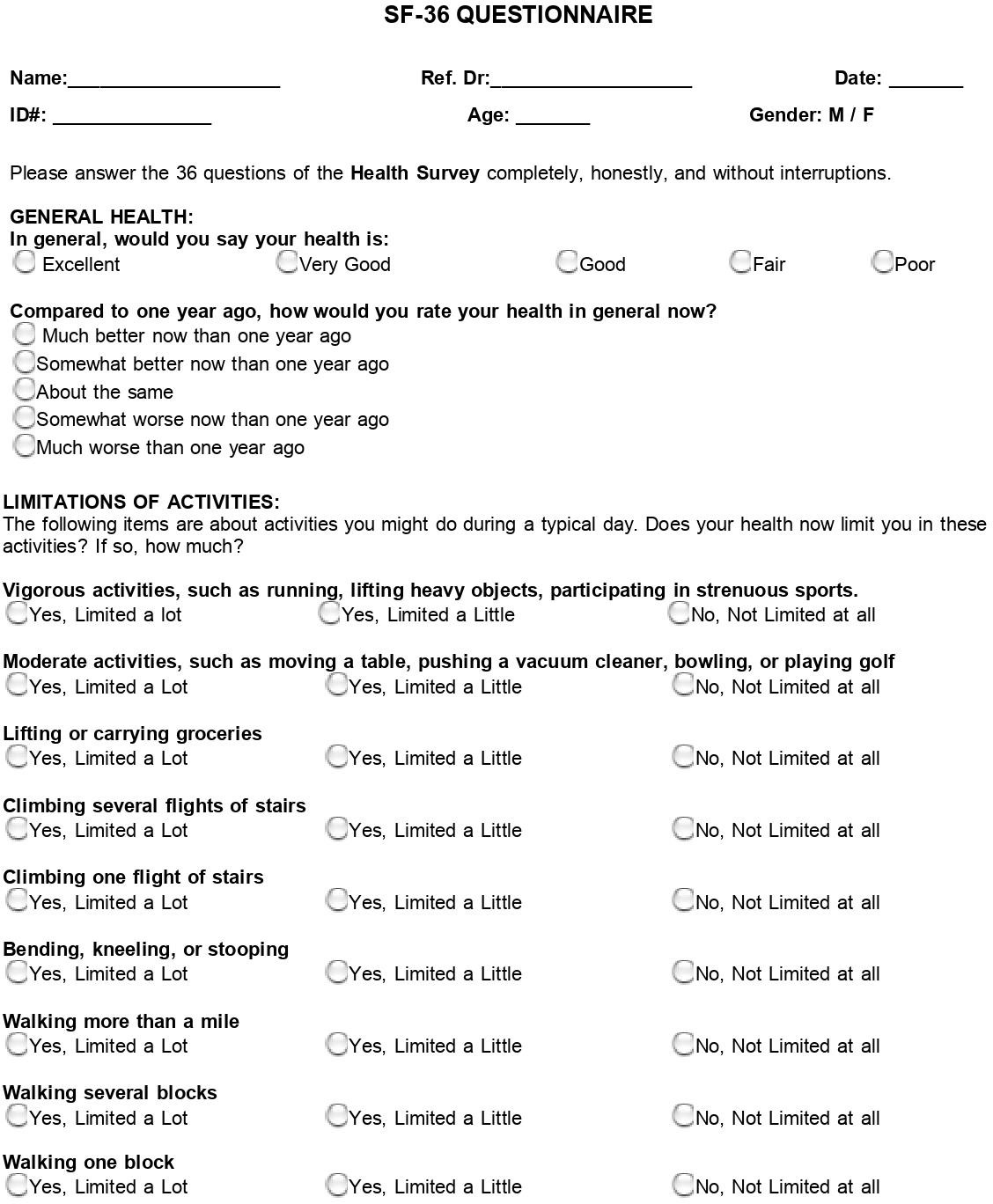


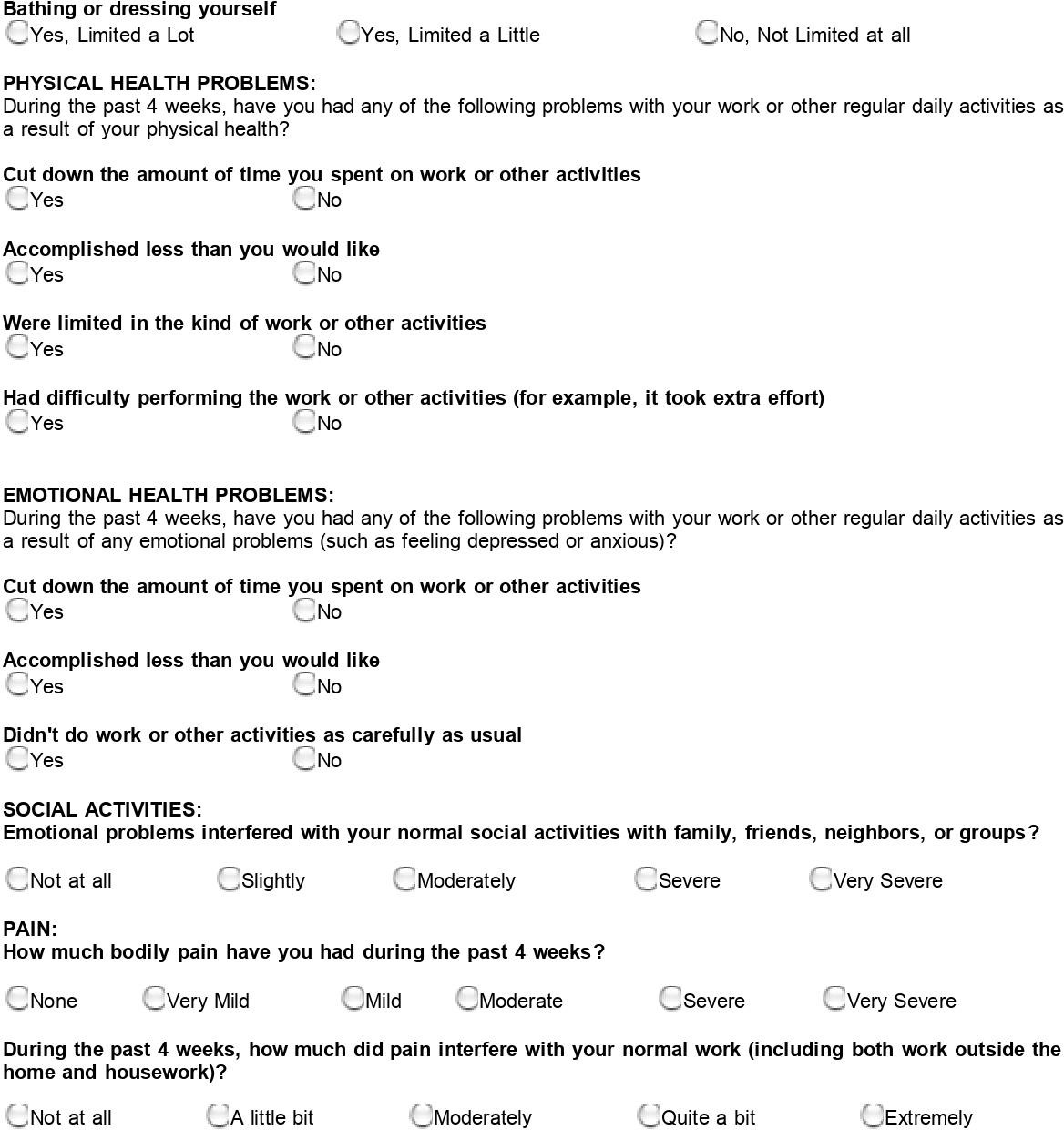


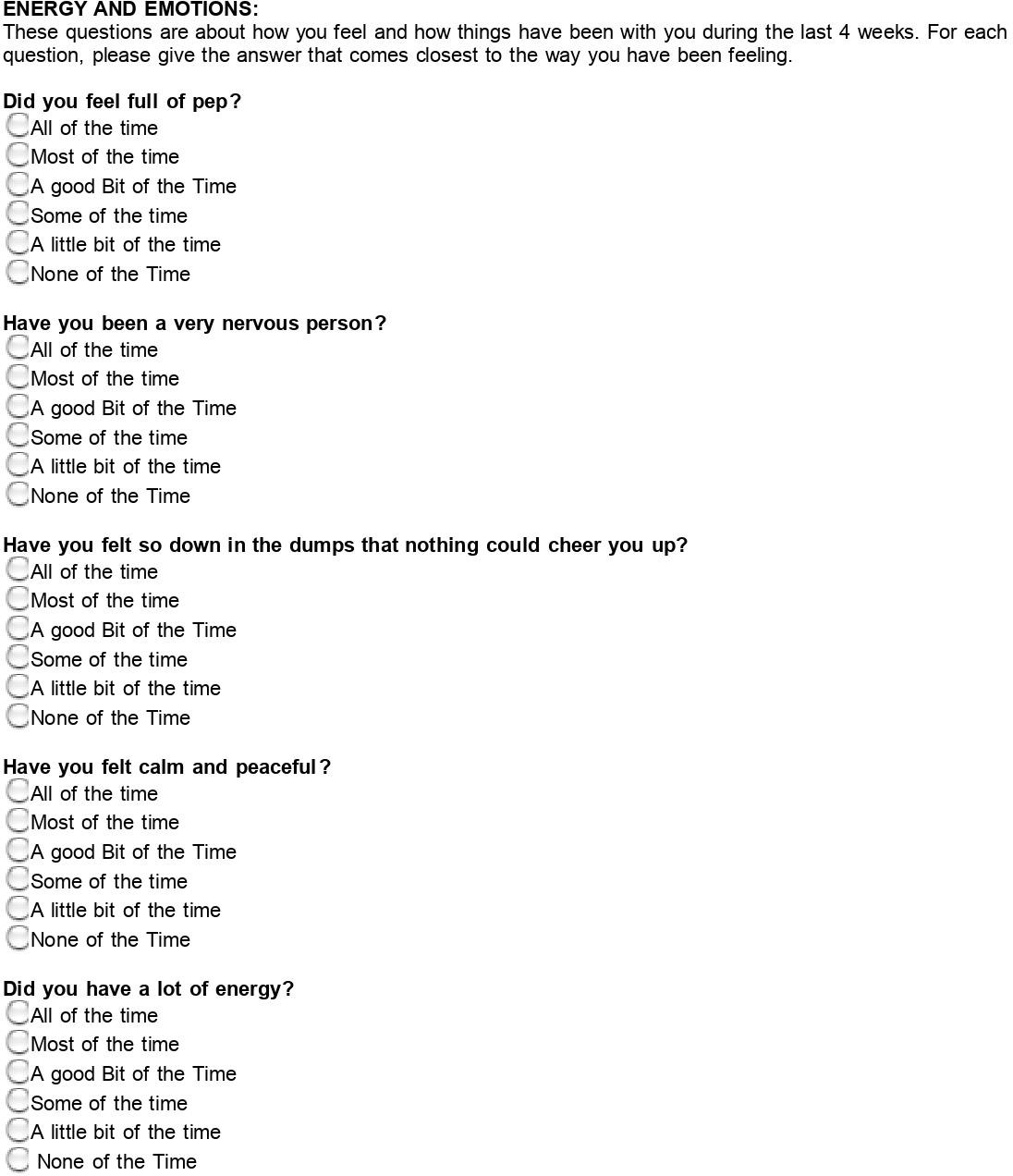


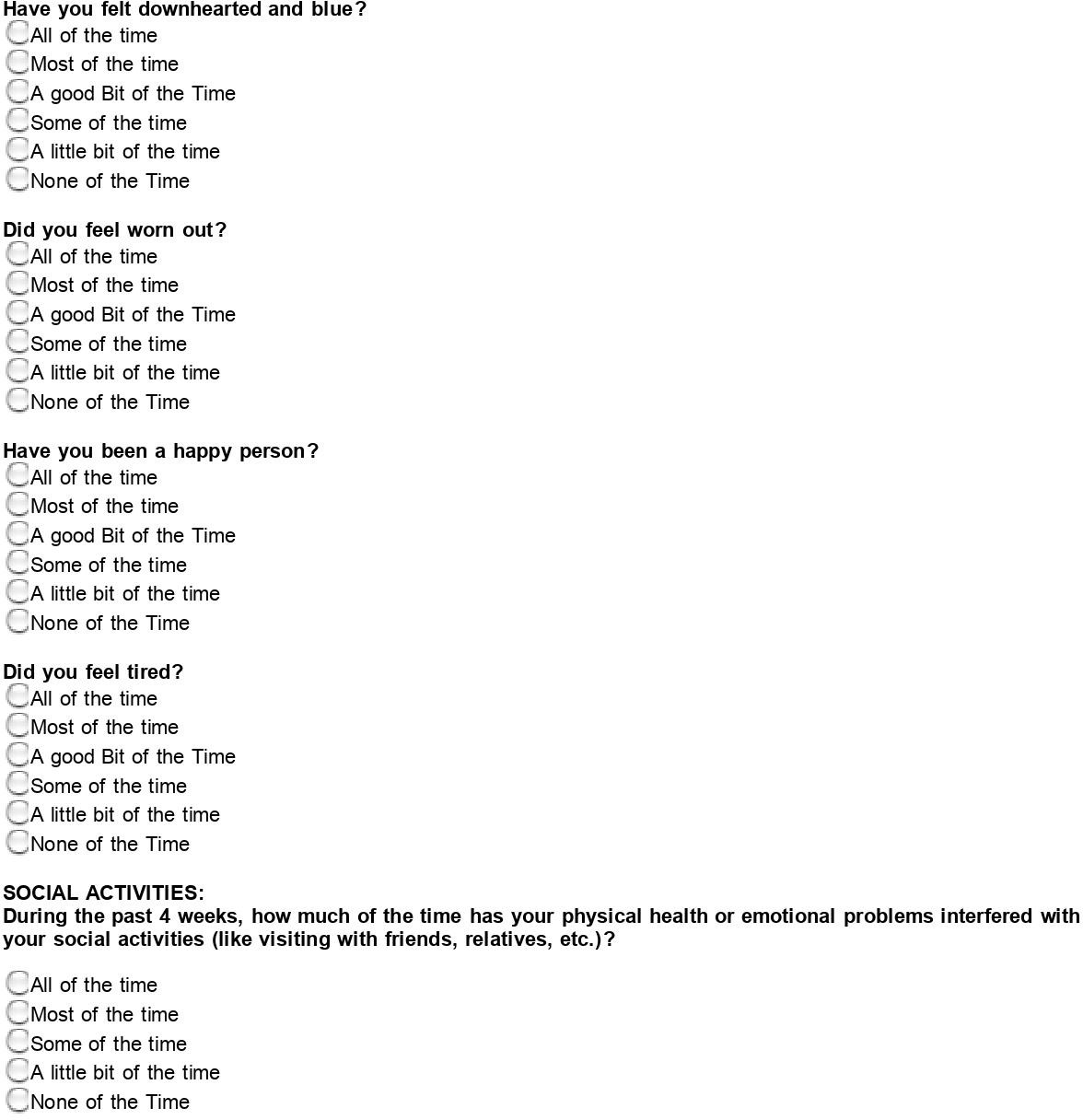


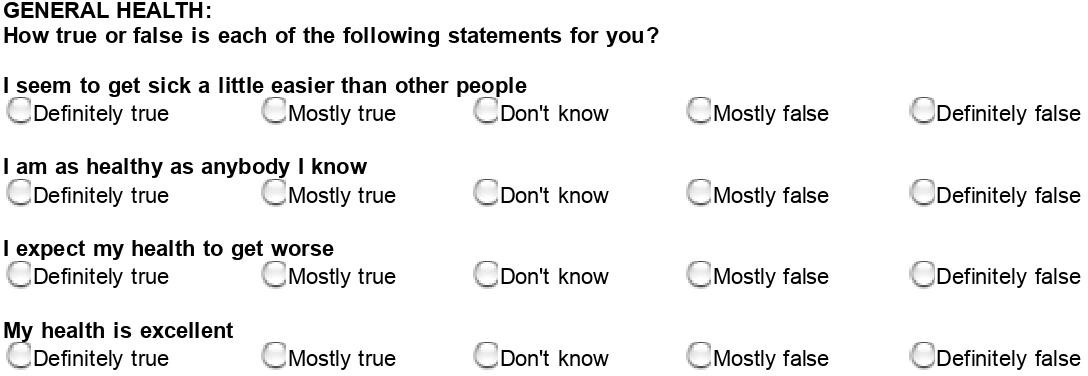


**ADHERENCE DATA: MORISKY-GREEN-LEVINE TEST (MGLT)**

**Instructions**: mark below with an “X” according to the treatment for UC

|  |  | **YES** | **NO** |
| --- | --- | --- | --- |
| **NON INTENTIONAL** | 1. Do you ever forget to talke your medicine? |  |  |
|  | 2. Are you careless at times about taking your medicine? |  |  |
| **INTENTIONAL** | 3. When you feel better do you sometimes stop taking your medicine? |  |  |
|  | 4. Sometimes if you feel worse when you take the medicine, do you stop taking it? |  |  |

1. According to MGLT, classify:
   1. Adherent, if answered NO to ALL questions
   2. Moderate adherence, if answered YES to 1 or 2 questions
   3. Low adherence, if answered YES to 3 or 4 questions

(9) NA

1. Non-adherence tipology according to MGLT: (1) Intentional (2) Non-intentional (3) Both (9) NA

**CLINICAL DATA**

4. CID: | | | | | , (9) NA

1. Weight (kg):
2. Height (m):

**UC CLINICAL ACTIVITY: LICHTIGER INDEX (LI)**

**INSTRUCTIONS**: Circle one answer for each symptom and the equivalent score.

| **SYMPTOMS** | **SCORE** |
| --- | --- |
| **Diarrhea (number of daily bowel movements)**  0-2  3 or 4  5 or 6  7 to 9  10 | 0  1  2  3  4 |
| **Nocturnal diarrhea**  No Yes | 0  1 |
| **Visible bleeding in stool (% of movements)**  0  <50  >50  100 | 0  1  2  3 |
| **Fecal incontinence**  No Yes | 0  1 |
| **Abdominal pain or cramps**  None Light Moderate  Severe | 0  1  2  3 |
| **General well being**  Perfect Very good Good Regular Bad  Terrible | 0  1  2  3  4  5 |
| **Abdominal tenderness**  None  Light and localized | 0  1 |

| Light to moderate and diffuse  Severe or rebound | 2  3 |
| --- | --- |
| **Need for antidiarrheal drugs**  No Yes | 0  1 |

1. Scores sum from the previous table:
2. Classify clinical activity according to total score:
   1. Remission (<4)
   2. Light/moderate activity (5-10)
   3. Severe colitis (>10)

(9) NA

PHARMACOTHERAPEUTIC DATA

1. In use for oral sulfassalazine 500mg: (1) yes (2) no (9) NA if yes, dose/day:
2. In use for oral mesalamine 400mg: (1) yes (2) no (9) NA if yes, dose/day:
3. In use for oral mesalamine 500mg: (1) yes (2) no (9) NA if yes, dose/day:
4. In use for oral mesalamine 800mg: (1) yes (2) no (9) NA if yes, dose/day:
5. In use for rectal mesalamine 250mg: (1) yes (2) no (9) NA if yes, dose/day:
6. In use for azathioprine 50mg:(1) yes (2) no (9) NA if yes, dose/day:
7. In use for oral cyclosporine 25mg: (1) yes (2) no (9) NA if yes, dose/day:
8. In use for oral cyclosporine 50mg: (1) yes (2) no (9) NA if yes, dose/day:
9. In use for oral cyclosporine 100mg: (1) yes (2) no (9) NA if yes, dose/day:
10. In use for oral cyclosporine 100mg/mL: (1) sim (2) no (9) NA if yes, dose/day:
11. Using medication for other conditions? (1) yes (2) no (9) NA if yes, answer items n° 20 and 21.
12. List medications currently in use for other diseases:

| MEDICATION  (generic name) | CONCENTRATIO N | PHARMACEUTI CAL FORM | ADMINISTRATIO N ROUTE | INDICATIO N | ATC CODE  (Level 5) | ATC CODE  (Level 3) |
| --- | --- | --- | --- | --- | --- | --- |
|  |  |  |  |  |  |  |
|  |  |  |  |  |  |  |
|  |  |  |  |  |  |  |
|  |  |  |  |  |  |  |
|  |  |  |  |  |  |  |
|  |  |  |  |  |  |  |

1. Observations:

#### Appendice 4 – Survey Questionnaire for final data collection

1. Participant identification code: | | | | |
2. Date (time 0): / /
3. Interviewer:

**QUALITY OF LIFE DATA (BRASIL SF 36)**

**Instructions**: this survey asks you about your health. This information will keep us informed of how you feel and how well you are able to carry out your activities of daily living. Answer each question by marking the answer as indicated. If you are unsure how to respond, please try to answer as best you can.


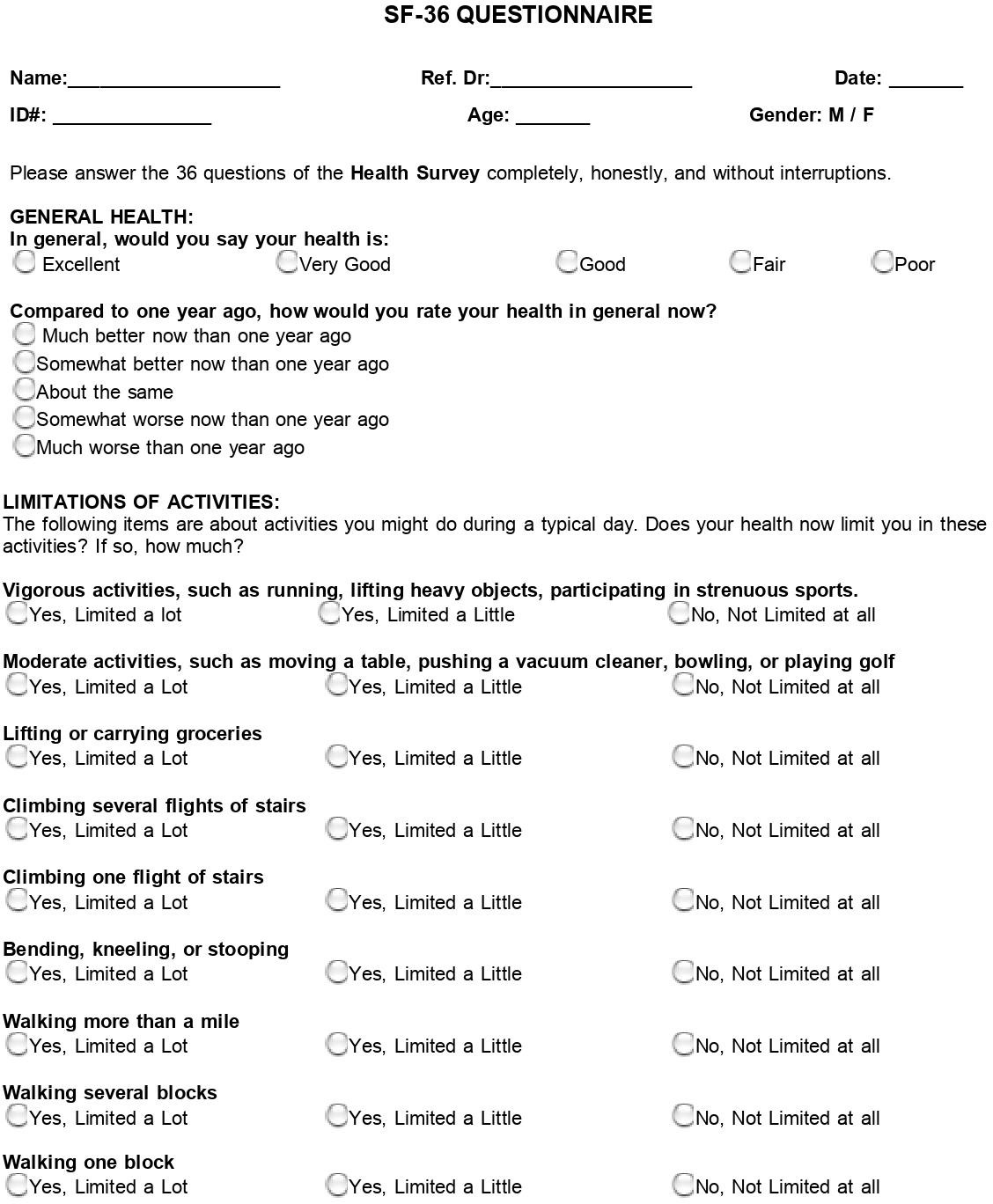


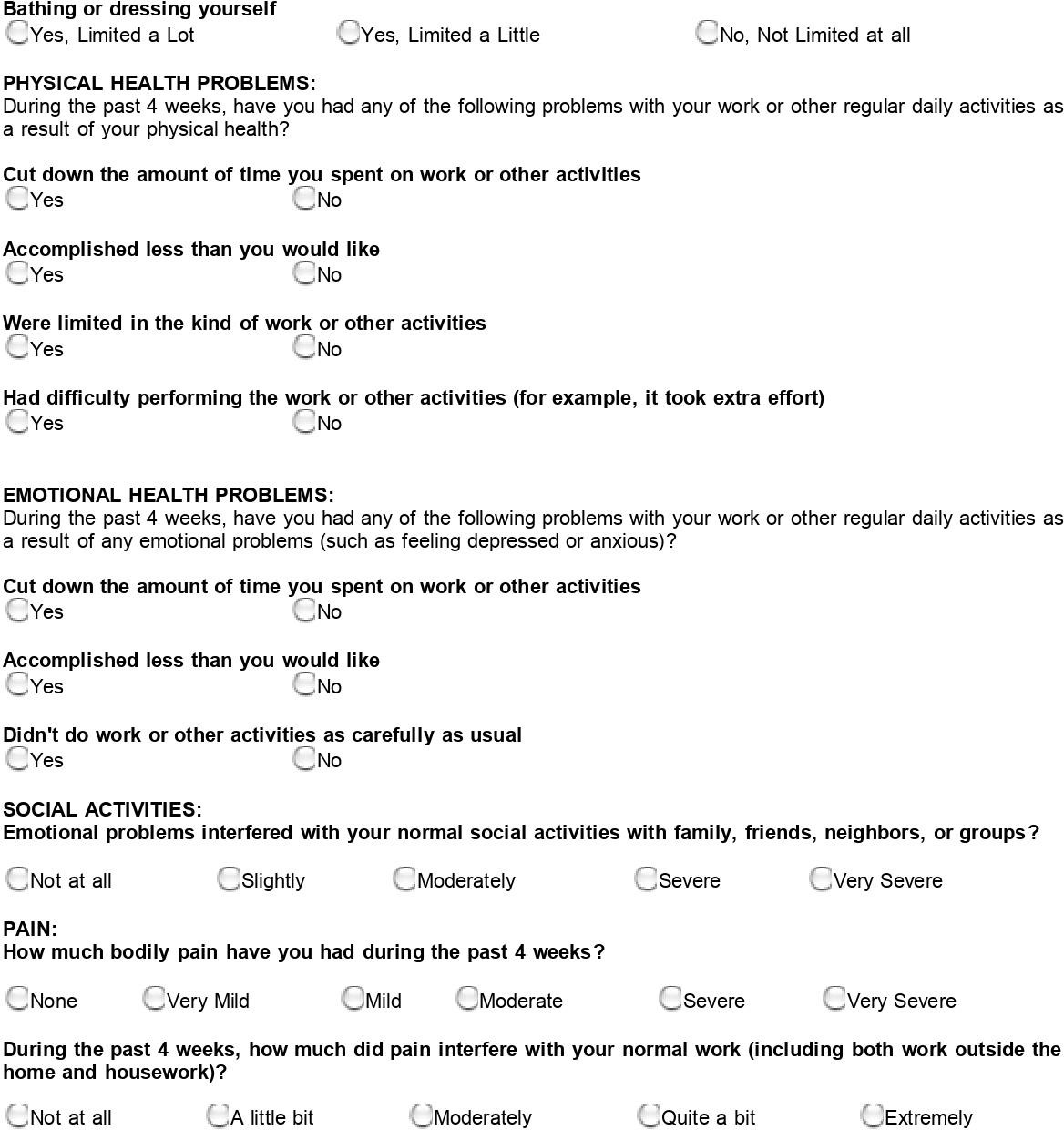


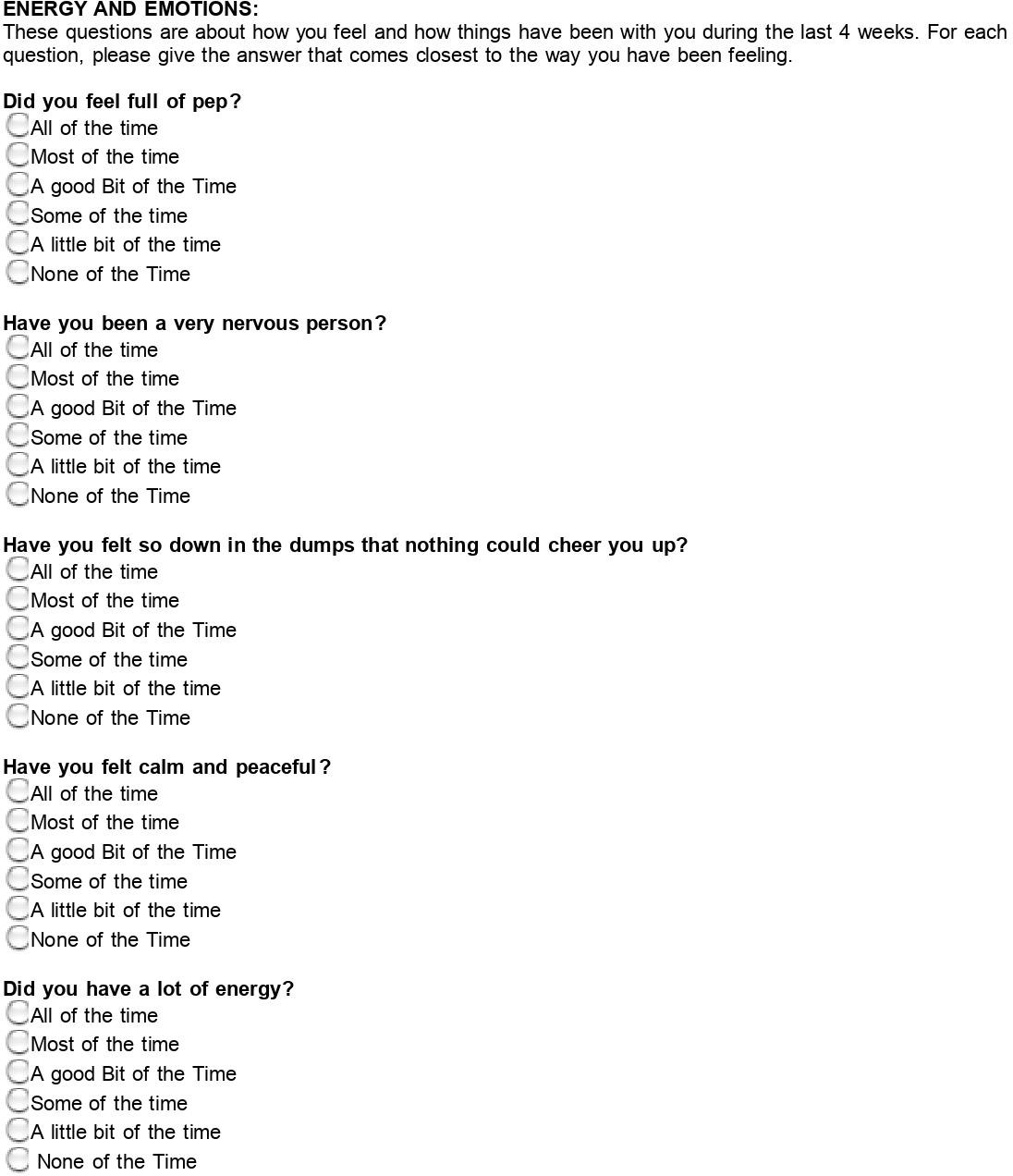


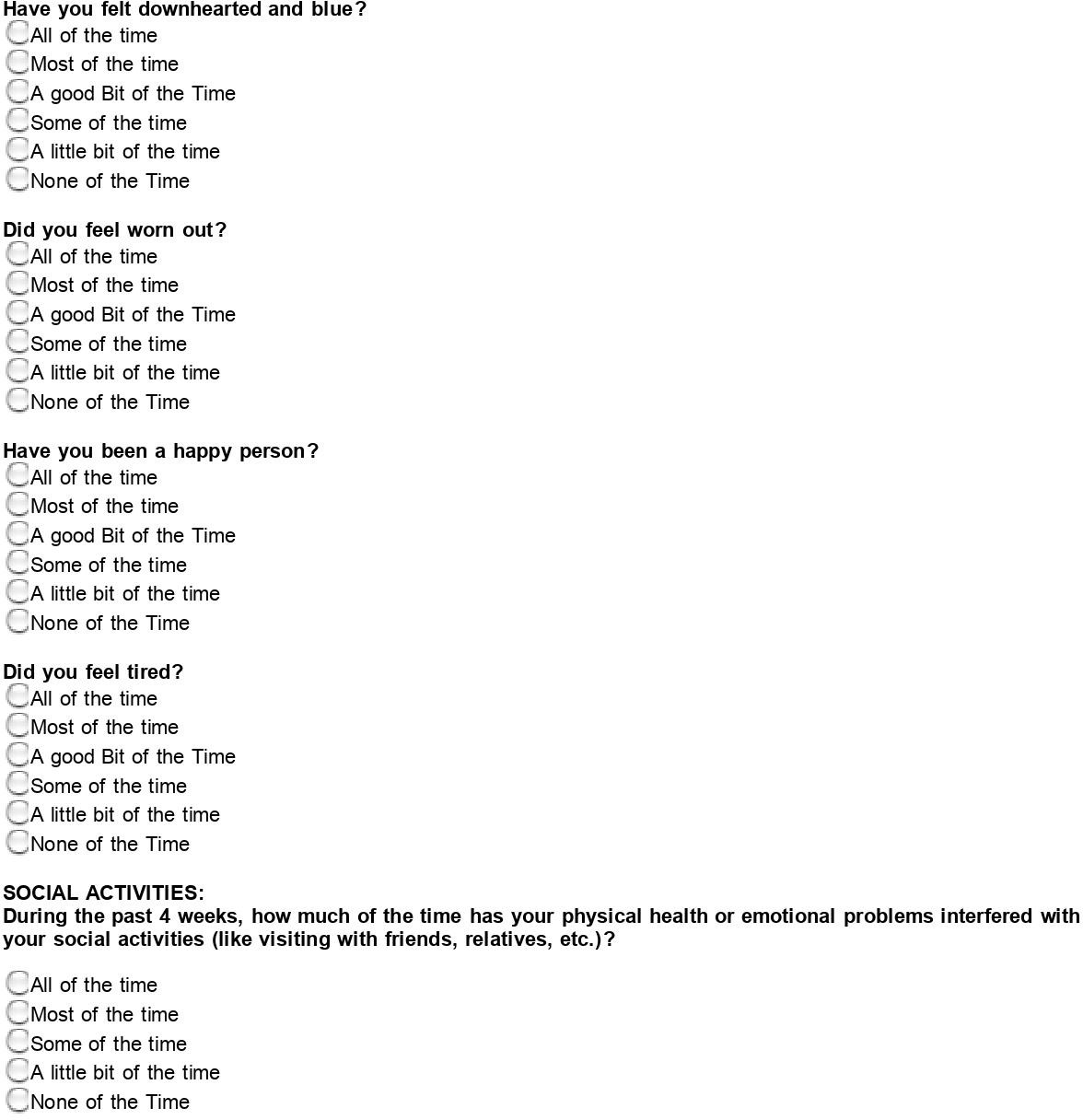


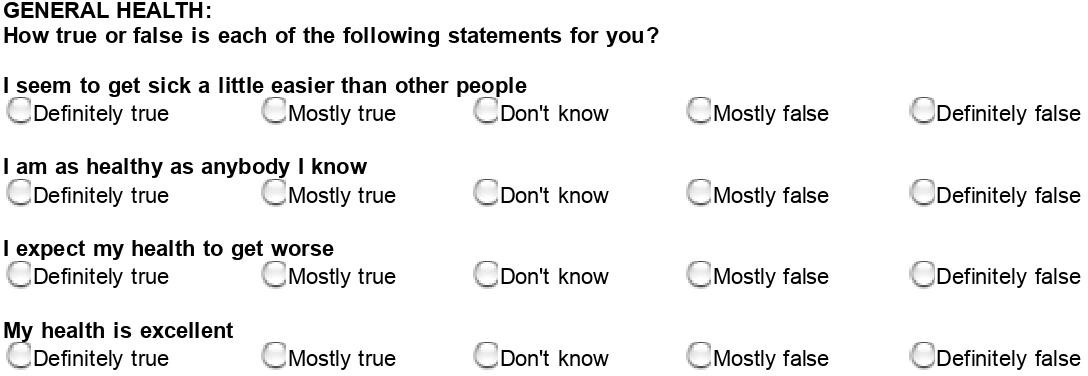


**ADHERENCE DATA: MORISKY-GREEN-LEVINE TEST (MGLT)**

### **Instructions**: mark below with an “X” according to the treatment for UC

|  |  | **YES** | **NO** |
| --- | --- | --- | --- |
| **NON INTENTIONAL** | 1. Do you ever forget to talke your medicine? |  |  |
|  | 2. Are you careless at times about taking your medicine? |  |  |
| **INTENTIONAL** | 3. When you feel better do you sometimes stop taking your medicine? |  |  |
|  | 4. Sometimes if you feel worse when you take the medicine, do you stop taking it? |  |  |

1. According to MGLT, classify:
2. Adherent, if answered NO to ALL questions
3. Moderate adherence, if answered YES to 1 or 2 questions
4. Low adherence, if answered YES to 3 or 4 questions

(9) NA

1. Non-adherence tipology according to MGLT: (1) Intentional (2) Non-intentional (3) Both (9) NA

**CLINICAL DATA**

1. Weight (kg):

**UC CLINICAL ACTIVITY: LICHTIGER INDEX (LI)**

**INSTRUCTIONS**: Circle one answer for each symptom and the equivalent score.

| **SYMPTOMS** | **SCORE** |
| --- | --- |
| **Diarrhea (number of daily bowel movements)**  0-2  3 or 4  5 or 6  7 to 9  10 | 0  1  2  3  4 |
| **Nocturnal diarrhea**  No Yes | 0  1 |
| **Visible bleeding in stool (% of movements)**  0  <50  >50  100 | 0  1  2  3 |
| **Fecal incontinence**  No Yes | 0  1 |
| **Abdominal pain or cramps**  None Light Moderate  Severe | 0  1  2  3 |
| **General well being**  Perfect Very good Good Regular Bad  Terrible | 0  1  2  3  4  5 |
| **Abdominal tenderness**  None  Light and localized  Light to moderate and diffuse Severe or rebound | 0  1  2  3 |

| **Need for antidiarrheal drugs**  No Yes | 0  1 |
| --- | --- |

1. Scores sum from the previous table:
2. Classify clinical activity according to total score:
   1. Remission (<4)
   2. Light/moderate activity (5-10)
   3. Severe colitis (>10)

(9) NA

#### Appendice 5 – Registration of the Intervention Program

1. Código de identificação do participante: | | | | |

### Instructions: record the date and mark with an “X” the intervention(s) performed:

| DATE | INTERVENTION PROGRAME | | | | | | | |
| --- | --- | --- | --- | --- | --- | --- | --- | --- |
|  | EDUCATIONAL INTERVENTIONS | | | | BEHAVIORAL INTERVENTIONS | | | |
|  | Video playback about IBD | Delivery of the UC brochure | Guidance on prescribed drugs for UC | Other guidance (on demand) | Delivery of Therapeutic Scheme | Delivery of the Pharmaceutical Guidance Table | Sending SMS Reminder Type | Sending SMS Motivationsl Type |
|  |  |  |  |  |  |  |  |  |
|  |  |  |  |  |  |  |  |  |
|  |  |  |  |  |  |  |  |  |
|  |  |  |  |  |  |  |  |  |
|  |  |  |  |  |  |  |  |  |
|  |  |  |  |  |  |  |  |  |
|  |  |  |  |  |  |  |  |  |
|  |  |  |  |  |  |  |  |  |
|  |  |  |  |  |  |  |  |  |
|  |  |  |  |  |  |  |  |  |
|  |  |  |  |  |  |  |  |  |
|  |  |  |  |  |  |  |  |  |

Observations:

#### Appendice 6 – Video Development Roadmap

1. – Information on IBD – definition; Signs and symptoms; phases (acute and remission); systemic commitment; fistulas;
2. – General information about the treatment – drug and non-drug;
3. – Adherence information – need to adhere to treatment even when symptoms are absent; importance of adherence to the prescribed therapeutic regimen; expected results with treatment adherence (improvement in signs/symptoms, improvement in quality of life).

#### Appendice 7 - Text for Video Preparation

Hi! Welcome!

I'm Raquel Rocha, a member of the Research Group at the Center for Gastroenterology at the Federal University of Bahia.

This video aims to inform about the main inflammatory bowel diseases, providing general guidelines on the importance of adherence to treatment.

Inflammatory Bowel Diseases are chronic inflammatory diseases of the digestive system, which can start from the mouth and continue through the esophagus, stomach, intestines and rectum, ending in the anus. Our defense system, also called the immune system, attacks the digestive system, resulting in inflammation and other consequences. There are 2 most well-known types of Inflammatory Bowel Diseases: Crohn's Disease and Ulcerative Colitis.

In Crohn's Disease, any part of the digestive system can become sick, from the mouth to the anus. This disease is characterized by inflammation, and there may be a narrowing of some portion of the intestine, which makes it difficult for the food bolus or feces to pass. In addition, in some cases, it can cause what we call a fistula. A fistula is an opening that occurs between two organs that would not normally be communicating, and this increases the risk of infection.

In ulcerative colitis, the inflammation is limited to the large intestine.

The main symptoms of Inflammatory Bowel Diseases in general are diarrhea, abdominal pain, weight loss, blood and mucus in the stool and fever. However, symptoms outside the intestine can occur, such as joint pain, skin, and eye problems.

It is important to say that these diseases have a characteristic of entering the acute phase of crises where the discomforts are present and also in the control phase, which we call remission, where the symptoms are absent or very mild. The treatment does not only consist of the use of medication, but other factors are also important, such as a healthy diet, physical activity, when released, and stress management. Often, those who have Inflammatory Bowel Diseases, when they enter the remission phase, that is, control, have so much improvement that they believe they are cured, however, as the disease is chronic, if you discontinue the medication and all other measures, you can return to have crises.

The objective of treatment is to keep the disease under control, but, in addition, often even with no symptoms, when tests are performed, inflammation is still present. This inflammation can be present without symptoms, so treatment should be continued to achieve healing.

As Inflammatory Bowel Diseases have no cure, but control, treatment must be done throughout life. Be sure to take your medicines and always take them at the indicated times, because the more correct the use, the better the result. Most drugs do not take effect immediately, so the importance of maintaining continuous use, because when you stop the drug, it is as if you are starting from scratch. In addition, it is important to say that only with treatment is it expected to have a favorable result. In addition, with proper treatment, it is expected to have a quality life, being able to return to routine activities.

So, take care of yourself, and any questions about the treatment, contact the team of health professionals to guide you. If you have any discomfort due to the use of your medications, talk to your doctor or pharmacist.

We hope this video has helped you understand a little more about Inflammatory Bowel Diseases, and the importance of treatment. You can access this video at any time, as it is freely accessible to all.

Until later!

## Appendice 8 – Educational Brochure (Front and Back)

#### Appendice 9 – Roadmap for Preparing the Educational Brochure

- 1. – RCU information – definition; Signs and symptoms; phases (acute and remission); systemic commitment;
  2. – General information about the treatment – drug and non-drug;
  3. – Adherence information – need to adhere to treatment even when symptoms are absent; importance of adherence to the prescribed therapeutic regimen; expected results with treatment adherence (improvement in signs/symptoms, improvement in quality of life).

#### Appendice 10 - Medicines for UC available at FIMAE

| **MEDICATION / DOSAGE** | **PHARMACEUTICAL FORM / PRESENTATION** | **ADMINISTRATION ROUTE** |
| --- | --- | --- |
| Azathioprine 50mg | Pill | Oral |
| Cyclosporina 25mg | Capsule | Oral |
| Cyclosporina 50mg | Capsule | Oral |
| Cyclosporina 100mg | Capsule | Oral |
| Cyclosporina 100mg/mL | Oral suspension | Oral |
| Mesalamine 250mg | Suppository | Rectal |
| Mesalamine 400mg | Pill | Oral |
| Mesalamine 500mg | Pill | Oral |
| Mesalamine 800mg | Pill | Oral |
| Sulfassalazine 500mg | Pill | Oral |

**Appendice 11 – Roadmap for Guidance on Medicines**

1. Generic name of the drug (and trade name, if any), dosage, presentation, pharmaceutical form, route of administration;
2. Main contraindications, precautions and common adverse effects;
3. Most frequent drug interactions (drug/drug, drug/food, drug/alcohol and drug/smoking);
4. Storage, conservation and validity conditions according to the manufacturer;
5. Guidelines during use (examples: suppository application technique, use of the measuring cup).

**Appendice 12 – Model and Example of Therapeutic Scheme**

| 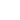TIME | MEDICATION NAME | QUANTITY / PHARMACEUTICAL FORM | OBSERVATIONS FOR USE | | | OTHER OBS. |
| --- | --- | --- | --- | --- | --- | --- |
|  |  |  | FASTING  (1h ANTES) | DURING MEAL | 2h AFTER MEAL |  |
| 8:00 | Azathioprine 50mg | 1 pill |  |  |  | Daily use |
| 8:00 | Mesalamine 250mg | 2 suppositories |  |  |  | Daily use, laying position |
| 20:00 | Mesalamine 250mg | 2 suppsitories |  |  |  | Daily use, laying position |
|  |  |  |  |  |  |  |
|  |  |  |  |  |  |  |
|  |  |  |  |  |  |  |
|  |  |  |  |  |  |  |
|  |  |  |  |  |  |  |
|  |  |  |  |  |  |  |
|  |  |  |  |  |  |  |
|  |  |  |  |  |  |  |
|  |  |  |  |  |  |  |

## Appendice 13 – Reminder Text (SMS)

“Hello, remember that on / / at : hours your return to FIMAE is scheduled.”

#### Appendice 14 – Texts (SMS) of the Motivational Type

“Take care of your health”

“Remember to take your medication regularly”

#### Attachment A – Pharmaceutical Guidance Table


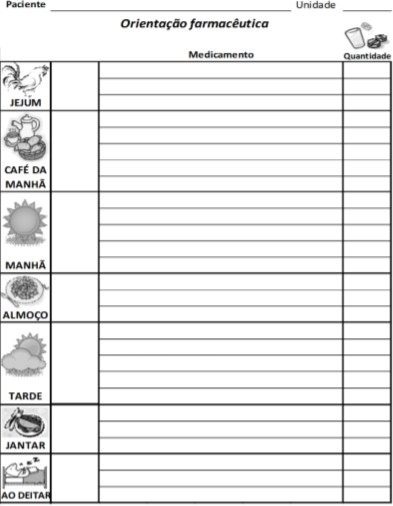


**Source: CANI, 2011.**
