# Supplementary material for: Impact of an intervention program on drug adherence in patients with ulcerative colitis: Randomized clinical trial
Source: PLoS One. 2023 Dec 27;18(12):e0295832. doi: 10.1371/journal.pone.0295832 (PMC10752503; doi:10.1371/journal.pone.0295832)
Supplement: S2 File — (DOC) [file pone.0295832.s002.doc]

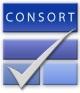


**Section/Topic**

**Item**

**NoChecklist item**

**Reported on page No**

CONSORT 2010 checklist of information to include when reporting a randomised trial*

# Title and abstract

**Introduction**

1a Identification as a randomised trial in the title S1, P1

1b Structured summary of trial design, methods, results, and conclusions (for specific guidance see CONSORT for abstracts) S1, P1

Background and 2a Scientific background and explanation of rationale objectives 2b Specific objectives or hypotheses

# Methods

Trial design 3a Description of trial design (such as parallel, factorial) including allocation ratio

3b Important changes to methods after trial commencement (such as eligibility criteria), with reasons Participants 4a Eligibility criteria for participants

4b Settings and locations where the data were collected

Interventions 5 The interventions for each group with sufficient details to allow replication, including how and when they were actually administered

Outcomes 6a Completely defined pre-specified primary and secondary outcome measures, including how and when they were assessed

6b Any changes to trial outcomes after the trial commenced, with reasons Sample size 7a How sample size was determined

7b When applicable, explanation of any interim analyses and stopping guidelines

| Randomisation:  Sequence | 8a | Method used to generate the random allocation sequence |
| --- | --- | --- |
| generation | 8b | Type of randomisation; details of any restriction (such as blocking and block size) |
| Allocation | 9 | Mechanism used to implement the random allocation sequence (such as sequentially numbered containers), |
| concealment  mechanism |  | describing any steps taken to conceal the sequence until interventions were assigned |

Implementation 10 Who generated the random allocation sequence, who enrolled participants, and who assigned participants to interventions

Blinding 11a If done, who was blinded after assignment to interventions (for example, participants, care providers, those

S2, P1

S2, P4

S3.2, P1

S3.16, P2

S3.4, P1

S3.1, P1

S3.11, P2

and S1_File

S3.14, P1-2

N/A

S3.13, P1

N/A

S3.7, P1

S3.7, P1

S3.7, P1

S3.8, P1

S3.10, P1-3

assessing outcomes) and how

11b If relevant, description of the similarity of interventions

Statistical methods 12a Statistical methods used to compare groups for primary and secondary outcomes 12b Methods for additional analyses, such as subgroup analyses and adjusted analyses

N/A

S3.15, P1-4

S3.15, P1-4

# Results

Participant flow (a 13a For each group, the numbers of participants who were randomly assigned, received intended treatment, and

| diagram is strongly |  | were analysed for the primary outcome | S4, P1 |
| --- | --- | --- | --- |
| recommended) | 13b | For each group, losses and exclusions after randomisation, together with reasons | S4, P1 |
| Recruitment | 14a | Dates defining the periods of recruitment and follow-up | S3.6, P1 and S3.11, P2 |
|  | 14b | Why the trial ended or was stopped | S3.13, P1 8 |
| Baseline data | 15 | A table showing baseline demographic and clinical characteristics for each group | S4.1, P1 |
| Numbers analysed | 16 | For each group, number of participants (denominator) included in each analysis and whether the analysis was  by original assigned groups | S4, P1 |

Outcomes and 17a For each primary and secondary outcome, results for each group, and the estimated effect size and its estimation precision (such as 95% confidence interval)

17b For binary outcomes, presentation of both absolute and relative effect sizes is recommended

Ancillary analyses 18 Results of any other analyses performed, including subgroup analyses and adjusted analyses, distinguishing pre-specified from exploratory

Harms 19 All important harms or unintended effects in each group (for specific guidance see CONSORT for harms)

**Discussion**

S4.2 and S4.3

S4.3, P2

N/A

S5, P13

Limitations 20 Trial limitations, addressing sources of potential bias, imprecision, and, if relevant, multiplicity of analyses

S5, P13

Generalisability 21 Generalisability (external validity, applicability) of the trial findings

Interpretation 22 Interpretation consistent with results, balancing benefits and harms, and considering other relevant evidence

# Other information

Registration 23 Registration number and name of trial registry

Protocol 24 Where the full trial protocol can be accessed, if available

Funding 25 Sources of funding and other support (such as supply of drugs), role of funders

N/A

S5, P1-14

S3.16, P1

S3, P1

S8, P1-4

*We strongly recommend reading this statement in conjunction with the CONSORT 2010 Explanation and Elaboration for important clarifications on all the items. If relevant, we also recommend reading CONSORT extensions for cluster randomised trials, non-inferiority and equivalence trials, non-pharmacological treatments, herbal interventions, and pragmatic trials. Additional extensions are forthcoming: for those and for up to date references relevant to this checklist, see [www.consort-statement.org.](http://www.consort-statement.org/)
